# Probing the Dynamics Process of High‐Entropy Alloy Catalysts: Fundamentals, Synthesis, and In Situ TEM Insights

**DOI:** 10.1002/advs.202514577

**Published:** 2025-09-30

**Authors:** Haider Ali, Yan Xu, Xiao Han, Yue Lin

**Affiliations:** ^1^ Hefei National Research Center for Physical Sciences at the Microscale University of Science and Technology of China Hefei Anhui P. R. China

**Keywords:** formation mechanism, high entropy alloys, in situ TEM, machine learning

## Abstract

Catalysts play a crucial role in advancing green and clean energy technologies for a sustainable future. Among the various catalysts reported, high‐entropy alloys (HEAs) stand out due to their superior stability and synergistic effects during catalytic processes, which arise from their complex composition and dynamic behavior. However, despite their promising potential, the incorporation of multiple elements (often more than four) with differing properties in HEA‐based catalysts complicates their growth and challenges their synthesis. Recent development of delicate synthetic strategies and theoretical understanding promotes the effective construction of HEAs. Advances of visible characterization techniques contribute to the cognition for the formation and evolution mechanisms of HEAs and further facilitate the accurate design. This review aims to provide a comprehensive overview of current synthesis strategies for HEA‐based catalysts while exploring their growth and evolution under real reaction conditions using in situ transmission electron microscopy (in situ TEM). First, the fundamental parameters, properties, and rational design principles of HEAs are examined to establish a detailed foundational understanding. Next, synthesis strategies for HEA‐based catalysts, highlighting the most frequently employed methods. The third section focuses on the application of in situ TEM in elucidating the atomic‐level mechanisms governing HEA formation. Finally, future opportunities are highlighted for designing next‐generation HEA catalysts.

## Graphical abstract

Schematic illustration of the basic properties, machine learning synthesis strategy, and formation under in situ TEM of HEAs.

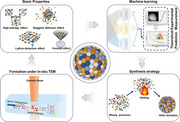

## 1 Introduction

Catalysis plays a vital role in advancing green energy solutions, mitigating pollution, and enabling the transformation of chemicals into more efficient and cleaner forms.^[^ ^1^ ^]^ These processes require high‐performance catalysts that combine stability, durability, abundant active sites, and cost‐effectiveness. In this context, alloy materials have emerged as particularly significant due to their tunable electronic and geometric structures. They are traditionally fabricated by incorporating small amounts of solute atoms into base metals,^[^ ^2^, ^3^ ^]^ where the limited compositional flexibility restricts the ability to finely regulate the electronic structure and optimize the adsorption energies of reaction intermediates, thereby hindering the maximization of catalytic activity.^[^ ^4^, ^5^ ^]^ These constraints have prompted the development of advanced materials with enhanced compositional diversity and entropy‐stabilized structures, leading to the emergence of high entropy alloys (HEAs).^[^ ^6^, ^7^ ^]^ HEAs depart from conventional alloy design by integrating multiple principal elements, typically more than four in near‐equimolar ratios. Their high configurational entropy promotes the formation of single‐phase solid solutions rather than complex intermetallic compounds. This paradigm shift has unlocked a vast, previously inaccessible design space, allowing for unprecedented control over composition and functionality.^[^ ^8^, ^9^, ^10^ ^]^ HEAs have since demonstrated exceptional properties, including resistance,^[^ ^11^ ^]^ to irradiation, enhanced mechanical strength,^[^ ^12^ ^]^ along with exceptional thermal and chemical stability.^[^ ^13^ ^]^ Notably, their catalytic and photothermal performances have opened new frontiers in sustainable energy technologies to environmental remediation,^[^ ^14^, ^15^ ^]^ and biomedical solutions.^[^ ^16^ ^]^

The atomic‐scale environment in HEAs differs fundamentally from traditional alloys, the random mixing of multiple elements produces unique electronic interactions.^[^ ^17^ ^]^ These materials exhibit inherent structural complexity across multiple scales. At the atomic level, the loss of perfect periodicity creates localized strain fields that create localized distortions in the crystal lattice, while nanoscale chemical ordering and clustering phenomena develop spontaneously. Such multi‐level heterogeneity creates effective barriers against dislocation movement, explaining the remarkable strength observed in many HEAs. Meanwhile, the diverse atomic configurations create a wide range of surface binding sites and an almost continuous spectrum of binding energies. This characteristic is particularly advantageous for catalysis, as it enables precise tuning of binding interactions to optimize reaction pathways.^[^ ^18^ ^]^ Additionally, HEAs demonstrate remarkable functional properties, including superior superconductivity, exceptional thermal stability, unique superparamagnetic behavior, and efficient hydrogen storage capabilities.^[^ ^19^, ^20^, ^21^, ^22^ ^]^

The properties of HEAs, from their entropy‐stabilized phases to their adjustable functional behavior, are fundamentally determined by their synthesis pathways. However, fabricating complex multi‐component systems into one phase presents distinct challenges, such as elemental segregation, phase control, and scalable production. Overcoming these obstacles requires a solid understanding of the underlying thermodynamic and kinetic principles, including configurational entropy (ΔS_mix_), atomic size mismatch (δ), and mixing enthalpy (ΔH_mix_), which guide the selection of element combinations that stabilize single‐phase solid solutions and suppress phase separation.^[^ ^23^ ^]^ Leveraging these principles, computationally guided synthesis enables the predictive design of HEAs tailored to specific catalytic reactions, minimizing reliance on conventional trial‐and‐error methods.^[^ ^24^ ^]^ To meet practical demands, advanced synthesis techniques have been developed to achieve controlled morphologies, compositional uniformity, and scalability. Recent progress in the step‐alloying method and liquid metal‐assisted synthesis has further enabled the production of nanoscale HEAs with tunable surface properties, enhancing their catalytic potential.^[^ ^25^, ^26^ ^]^ Complementing these advances, in situ characterization techniques, particularly in situ transmission electron microscopy (in situ TEM), provide real‐time atomic‐scale insights into HEAs formation, revealing nucleation pathways, phase transitions, and stability thresholds.^[^ ^27^ ^]^ Together, the integration of theoretical modeling, scalable synthesis, and atomic‐resolution validation offers a comprehensive framework for the rational engineering of HEA catalysts with optimized activity, durability, and cost‐efficiency for real‐world applications.

**Scheme 1:**
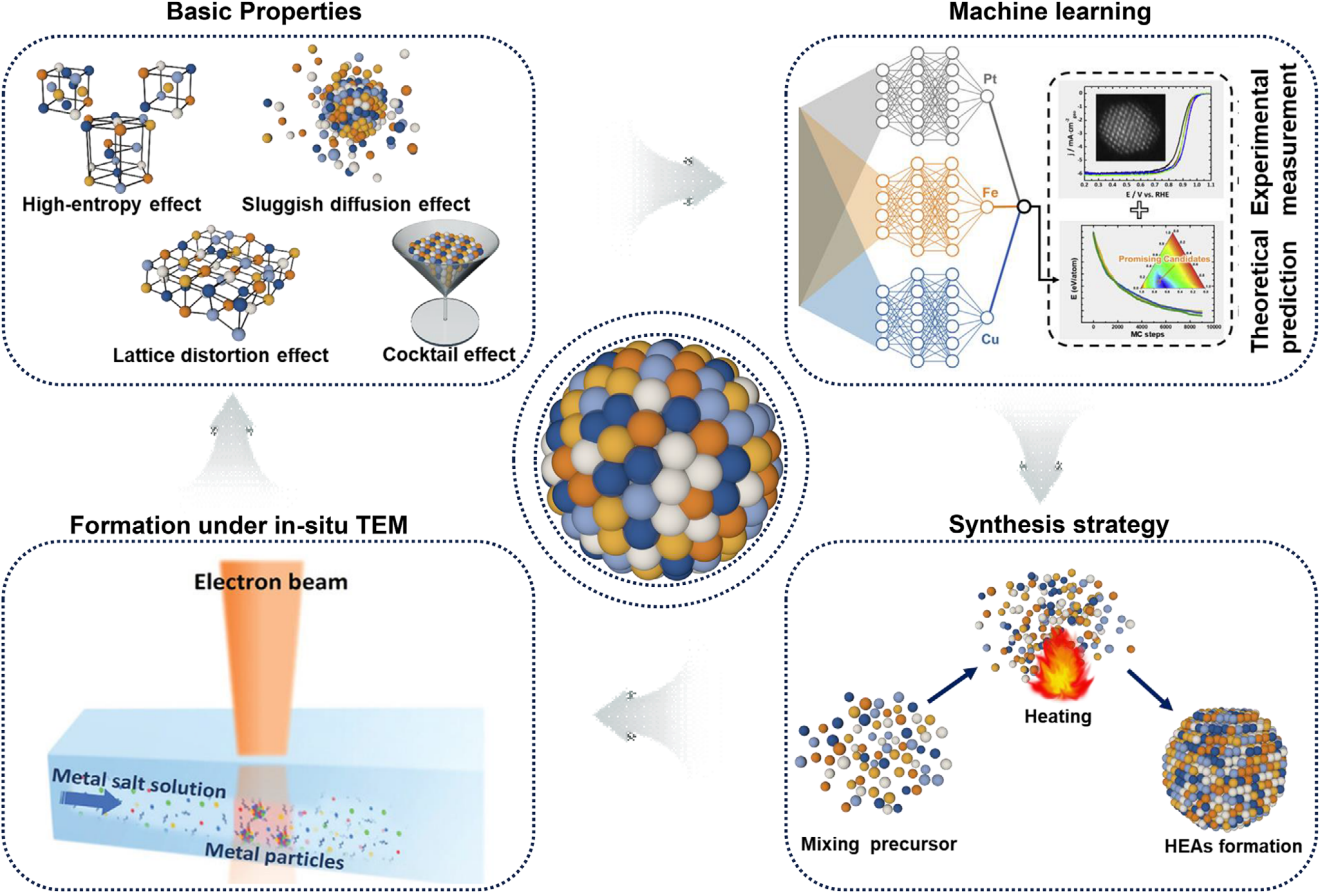
Schematic illustration of the basic properties, machine learning. Reproduced with permission.^[^ ^28^ ^]^ Copyrights 2021, Elsevier, synthesis strategy and formation under in situ TEM of HEAs. Reproduced with permission.^[^ ^29^ ^]^ Copyright 2024, Wiley‐VCH GmBH.

In this review, we summarize HEAs across four key sections (**Scheme** **1**). First, we establish the design principles of HEAs by introducing five fundamental parameters as theoretical descriptors to guide element selection and evaluate phase stability, alongside advanced modification strategies such as crystal structure engineering and surface modification to optimize their catalytic properties. Next, we provide a systematic evaluation of the most frequently employed synthetic methodologies, offering a comparative framework to facilitate the rational selection of appropriate strategies for HEA catalyst design. We then highlight recent advances in in situ TEM studies, which uncover the formation, growth, and evolution mechanisms of HEAs at the atomic level under realistic conditions, and further discuss their prospective role in guiding future HEA research. Finally, we conclude with emerging perspectives and directions in the field. This review aims to deliver meaningful insights into the synthesis, design principles, and atomic‐scale engineering of HEA‐based catalysts, thereby supporting advanced catalytic applications and inspiring further innovation in this promising material class.

## 2 Fundamentals of High‐Entropy Alloys

### 2.1 Basic Parameters

This section explores the essential concepts governing the development and stability of HEAs. HEAs emerge from a multi‐principal element design approach, resulting in unique thermodynamic behavior, atomic arrangements, and material characteristics. Based on thermodynamic principles and empirical evidence, five essential elements significantly influence the production of HEAs, that is, mixing entropy, mixing enthalpy, atomic size disparity, valence electron concentration, and electronegativity variation. These factors are fundamentally connected to the alloys chemical composition and are crucial in influencing the formation mechanisms and overall stability of HEAs.

#### 2.1.1 Mixing Entropy (ΔS_mix_)

**Figure 1:**
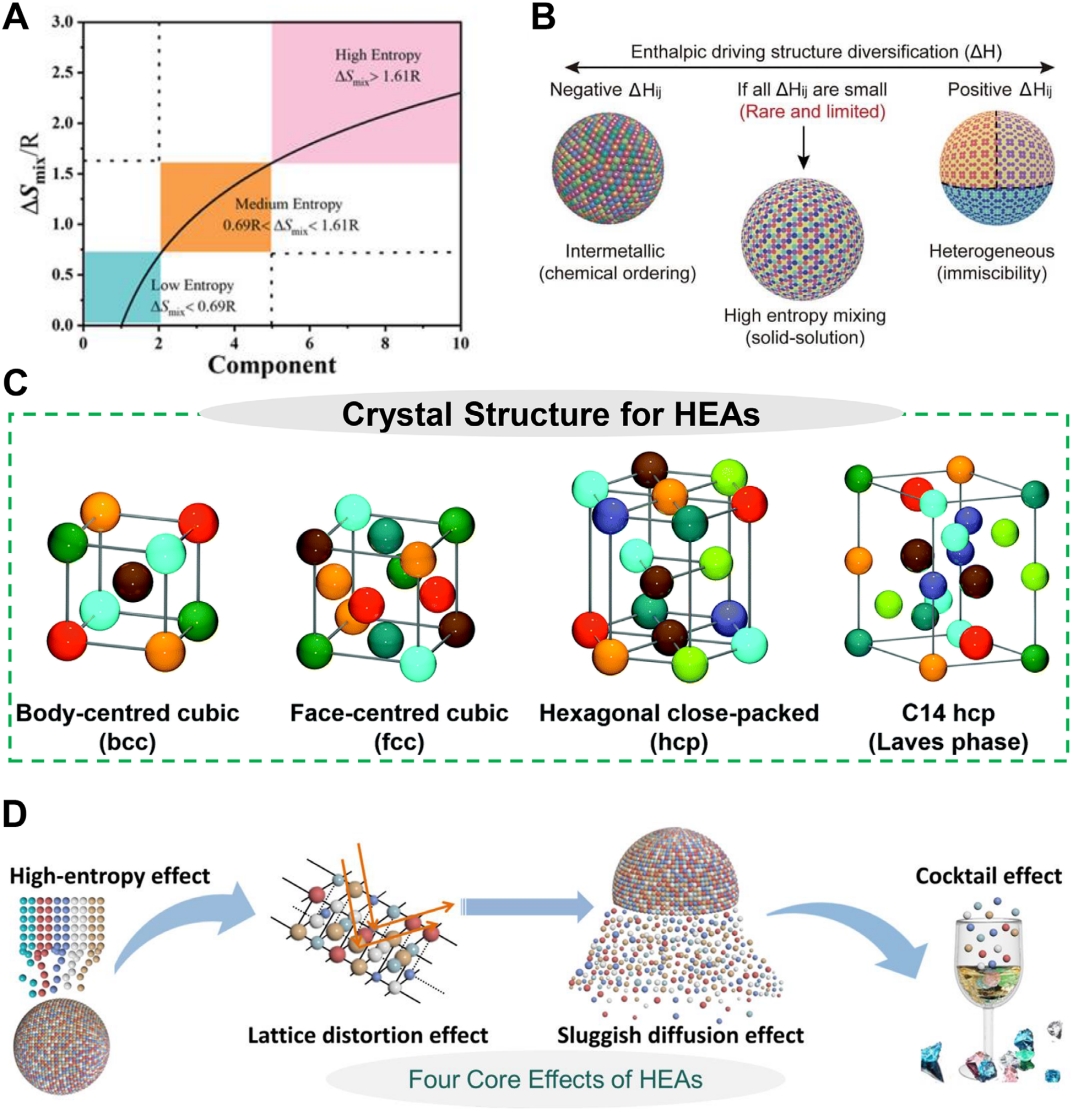
Key thermodynamic and structural properties HEAs. A) Influence of configurational entropy (ΔS_mix_). Reproduced with permission.^[^ ^30^ ^]^ Copyright 2022, Wiley‐VCH GmbH and B) Role of mixing enthalpy (ΔH_ij_) governing atomic interaction in HEAs. Reproduced with permission.^[^ ^24^ ^]^ Copyright 2022, American Association for the Advancement of Science. C) Predominant crystal structures (FCC, BCC, HCP, or Laves phases) observed in single‐phase HEAs. Reproduced with permission.^[^ ^30^ ^]^ Copyright 2022, Wiley‐VCH GmbH. D) The four fundamental core effects (high‐entropy, sluggish diffusion, severe lattice distortion, and cocktail effect) defining HEA behavior. Reproduced with permission.^[^ ^5^ ^]^ Copyright 2024, American Association for the Advancement of Science.

The configurational entropy of HEA NPs escalates with the growth in the number of constituent elements, acting as a crucial thermodynamic stimulus that facilitates the development of a single‐phase solid solution (**Figure** **1**A).^[^ ^30^ ^]^ This increased entropy improves the system's overall stability. The theoretical value of the ideal mixing entropy ΔS_mix_ for high‐entropy alloys can be obtained using Boltzmann's statistical principle.

(1) $$\Delta S_{mix}=-R\sum n_{i}\ln n_{i}$$

Here, “R” indicates the universal gas constant (8.314 Jmol^−1^K^−1^), while n_1_, n_2_, … represent the atomic percentages of each constituent. For equi‐atomic compositions in which each element is present in the same proportions (n_1_ = n_2_ = n_3_ = …), the equation is simplified to.

(2) $$\Delta S_{mix}=-R\ln n$$

where n represents the total number of elements in the alloy. Consequently, ΔS_mix_ provides approximate values of 1.39R, 1.61R, and 1.79R for equi‐atomic systems including 4, 5, and 6 elements, respectively. An increased configurational entropy not only aids in stabilizing the solid solution phase but also mitigates the creation of ordered phases and phase separation.

#### 2.1.2 Enthalpy (∆H_mix_)

In thermodynamics, the Gibbs free energy of mixing (ΔG_mix_) determines whether a single‐phase solid solution or competing phases will develop. It is described by this equation

(3) $$\Delta G_{mix}=\Delta H_{mix}-T\Delta S_{mix}$$

Here, T represents the absolute temperature. ∆H_mix_ and ΔS_mix_ denote the mixing enthalpy and the configurational entropy, respectively. Consequently, understanding and evaluating the ∆H_mix_ is crucial for predicting phase formation behavior in HEAs.

The mixing enthalpy represents the chemical interaction between elements and can be estimated using the following expression:

(4) $$\Delta H_{mix}=\sum_{i=1.i\neq j}^{n}4\Delta H_{ij}^{mix}x_{i}x_{j}$$

Here, x_i_ or x_j_ is the atomic percentage of the i^th^ or j^th^. $\Delta H_{ij}^{mix}$ represents the mixing enthalpy of the atom pair between the i^th^ and j^th^ components.

A positive mixing enthalpy signifies repulsive interactions among elements, perhaps facilitating phase separation or elemental segregation. A larger negative mixing enthalpy indicates stronger attractive contacts, promoting the development of intermetallic compounds (Figure 1B).

#### 2.1.3 Atomic Size Difference

Besides thermodynamic variables, the disparity in atomic size is a crucial topological parameter that substantially influences the structural stability of HEAs. This parameter can be mathematically represented by the subsequent equation.

(5) $$\begin{array}{c} \delta =\sqrt{\sum_{i=1}^{n}x_{i}\left(1-\frac{r_{i}}{\bar{r}}\right)}\\ \bar{r}=\sum_{i=1}^{n}x_{i}r_{i} \end{array}$$

Here, $\bar{r}$ is the average radius of all elements, r_i_ and x_i_ represent the atomic radius and atomic fraction of the i^th^ element, respectively.

A significant atomic size discrepancy results in considerable lattice distortion, potentially disrupting the solid solution structure and initiating phase transitions. Consequently, regulating atomic size variation is essential for preserving the structural integrity and phase stability of HEAs.

#### 2.1.4 Valence Electron Concentration (VEC)

The valence electron denotes the electrons capable of engaging with other atoms to establish chemical bonds. The VEC of HEAs influences the phase structure via modulating bonding and stacking features, which can be computed as follows.

(6) $$VEC=\sum_{i=1}^{n}x_{i}{\left(VEC\right)}_{i}$$

where (VEC)_i_ represents the VEC of the i^th^ component, x_i_ is the percentage of atoms of the i^th^ component.

#### 2.1.5 Electronegativity

Electronegativity reflects an atoms tendency to attract electrons in a chemical bond. In the context of HEAs, the difference in Pauling electronegativity among constituent elements can be calculated using the following expression

(7) $$\begin{array}{c} \Delta \chi_{Pauling}=\sqrt{\sum_{i=1}^{n}x_{i}{\left(\chi_{i}-\chi_{avg}\right)}^{2}}\\ \chi_{avg}=\sum_{i=1}^{n}x_{i}\chi_{i} \end{array}$$

where x_i_ is the atomic percentage and χ_*i*_ describes the Pauling electronegativity of the i^th^ component.

The ΔS_mix_ of HEAs increases with the number of constituent elements, serving as a key thermodynamic driver that promotes the homogeneous distribution of diverse metallic atoms, particularly at elevated temperatures. However, this entropy‐driven stabilization competes with the enthalpy of formation (ΔH_ij_) between dissimilar elements, which governs the bonding interactions and varies significantly depending on atomic size disparities, electronic structure, and electronegativity differences.^[^ ^31^ ^]^ While high configurational entropy generally favors the formation of single‐phase solid solutions, it does not guarantee their stability.

The final phase outcome depends on the delicate balance between ΔS_mix_ and the enthalpiccontributions of the constituent elements. For instance, element pairs with strongly positive ΔH_ij_ values (e.g., Au─Ni or Au─Pt), indicative of repulsive interactions, tend to exhibit limited miscibility, often resulting in phase separation. Conversely, systems with highly negative ΔH_ij_ (e.g., Pt─Ni or Pt─Sn), reflecting strong attractive interactions, favor atomic ordering and the precipitation of intermetallic compounds.^[^ ^31^ ^]^

As summarized in **Table** **1**, the intrinsic physicochemical properties of individual elements, such as atomic radius, electronegativity, and reduction potential, play a crucial role in determining the crystal structure and stability of HEAs. These parameters directly influence lattice distortion, phase selection (face‐centered cubic (FCC), body‐centered cubic (BCC), hexagonal closed‐packed (HCP), or Laves phases), and possible phase transitions (Figure 1C).^[^ ^32^, ^33^ ^]^ These parameters critically govern HEA formation by modulating atomic packing and bonding behavior.

**Table 1:** The different physicochemical properties of the individual elements and their salts.

Metals	Atomic Radius [Å]	Electronegativity	Melting point [K]	Room‐T structure	Metals salts	Reduction potential [V]
Pt	1.39	2.28	2041	FCC	H_2_PtCl_6_	0.68, 0.73, 1.18
Pd	1.37	2.20	1828	FCC	PdCl_2_	0.95
Rh	1.34	2.28	2236	FCC	RhCL_3_	0.76
Ru	1.34	2.2	2527	HCP	RuCl_3_	0.45
Ir	1.36	2.20	2720	FCC	IrCl_3_	1.16
Fe	1.26	1.83	1811	BCC	FeCl_3_	0.77, ‐0.44
Co	1.25	1.88	1768	HCP	CoCl_2_	−0.28
Ni	1.24	1.91	1728	FCC	NiCl_2_	−0.25
Au	1.44	2.54	1337	FCC	HAuCl_4_	1.5
Cu	1.28	1.90	1358	FCC	CuCl_2_	0.34
Cr	1.28	1.66	2180	BCC	CrCl_3_.6H_2_O	−0.74
Mn	1.27	1.55	1519	BCC	MnCl_2_	−1.18
W	1.39	2.36	3695	BCC	WCl_6_	−0.12
Mo	1.39	2.16	2896	BCC	MoCl_5_	−0.15
Sn	1.40	1.96	505	Tetragonal	SnCl_2_	−0.14
Ti	1.47	1.54	1941	HCP	TiCl_4_	−1.37, −1.63
Zr	1.60	1.33	2128	HCP	ZrCl_4_	−1.45
Hf	1.59	1.3	2506	HCP	HfCl_4_	−1.72
V	1.34	1.63	2183	BCC	VCl_3_	0.34, −0.26, −1.13
Nb	1.46	1.6	2750	BCC	NbCl_5_	−1.10

### 2.2 The Four Core Effects

HEAs revolutionize conventional alloy development by integrating multiple principal elements in approximately equal atomic ratios, departing from the limitations of binary and ternary systems. This multifaceted method yields distinctive microstructures and characteristics, determined by a confluence of fundamental variables. HEAs are differentiated from conventional alloys by several fundamental properties, including the high‐entropy effect, lattice distortion, sluggish atomic diffusion, and the cocktail effect (Figure 1D).^[^ ^34^ ^]^ These features are crucial for advancing the field of HEAs, pushing materials science boundaries, and unlocking new possibilities in advanced materials research.

#### 2.2.1 The High Entropy Effect

The high‐entropy effect plays a crucial role in the behavior of HEAs by suppressing the formation of brittle, stoichiometric intermetallic compounds and inhibiting phase separation. Instead, it promotes the formation of solid solution phases,^[^ ^35^ ^]^ this effect significantly reduces the number of phases or crystal structures that form, defying the predictions of the Gibbs phase rule under constant pressure.^[^ ^34^ ^]^

The Gibbs phase rule is expressed as:

(8) P+F=C+1

P is the number of phases, F is the number of degrees of freedom, and C is the number of parts in the system.

The high‐entropy effect makes the configurational entropy higher, which makes the microstructure of HEAs less complex and allows the elements to interact with each other in a way that benefits them all. This makes the material stronger and more flexible, mostly through solution hardening mechanisms.^[^ ^36^, ^37^ ^]^ Also, the rise in mixing entropy helps to fill in the gaps in miscibility between different elements, which changes the composition and improves the electrocatalytic performance.^[^ ^38^ ^]^ The high‐entropy effect helps to stabilize HEAs thermodynamically, which is very helpful for getting outstanding catalytic durability during electrocatalysis.

#### 2.2.2 The Lattice Distortion Effect

HEAs often comprise solid solution phases that integrate several primary constituents. Each atom functions as a solute, occupying sites within a chemically intricate matrix. Consequently, atoms are prone to displace from their optimal lattice positions due to the effects of adjacent atoms with differing atomic sizes and asymmetric bonding properties, resulting in localized lattice distortions.^[^ ^39^, ^40^ ^]^ Lattice distortions significantly affect many material properties by generating local strain fields and interrupting the long‐range periodicity of the crystal lattice. These distortions affect both mechanical properties, such as enhanced hardness and altered ductility, and functional attributes, including electrical and thermal conductivity.^[^ ^15^ ^]^ The non‐equilibrium characteristics of a deformed lattice might reduce the energy barriers linked to the adsorption and activation of reactant molecules, which is especially advantageous in electrocatalytic reactions.^[^ ^41^ ^]^ The interaction of elevated configurational entropy with lattice distortion produces a synergistic effect that improves the structural stability, catalytic efficiency, and long‐term durability of HEAs in practical operational environments. These characteristics render HEAs exceptionally appealing for energy conversion and catalytic applications.

#### 2.2.3 The Sluggish Diffusion Effect

The prolonged diffusion effect characterizes HEAs, arising from the intricate local atomic environments created by the random arrangement of various elements inside a single‐phase lattice structure.^[^ ^34^ ^]^ Unlike traditional binary or ternary alloys, HEAs display a diverse range of atomic arrangements and bonding interactions. This diversity results in discrepancies in local potential energy landscapes and diffusion rates at certain lattice points.^[^ ^42^ ^]^ When atoms endeavor to move into vacancies, the diverse chemical environment can create a trapping effect, considerably obstructing atomic movement and diminishing diffusion rates.^[^ ^14^ ^]^ The inhibition of atomic mobility is intricately associated with the lattice distortion effect, wherein discrepancies in atomic radii and chemical affinities lead to significant deformation of the crystal lattice. Such distortions increase the energy barriers for atomic migration, hence exacerbating the sluggish diffusion behavior.^[^ ^43^ ^]^ The effect is especially advantageous during HEA synthesis, as they facilitate the preservation of nanocrystalline structures by constraining surface and bulk atom diffusion. The limited atomic mobility facilitates the regulation of microstructural evolution and improves the thermal and phase stability of HEAs, particularly in harsh or corrosive conditions. Thus, slow diffusion is crucial for the long‐term durability and functional reliability of HEAs in electrocatalytic and heterogeneous catalytic applications.

#### 2.2.4 The Cocktail Effect

The cocktail effect in HEAs refers to the distinctive synergistic interactions among various primary components, leading to material qualities that greatly exceed those anticipated by merely summing the contributions of individual constituents. This phenomenon is inherently linked to other essential characteristics of HEAs, specifically, elevated configurational entropy, lattice distortion, and sluggish diffusion, which collectively promote the development of a chemically disordered yet thermodynamically stable solid solution structure.^[^ ^44^ ^]^ Diverse atomic environments and bonding types result in non‐linear improvements in material properties, including diminished energy barriers for chemical reactions and expedited reaction rates in catalytic systems. These intricate interactions also affect the electrical structure and provide precise regulation of geometric configurations, both of which are essential for enhancing electrocatalytic efficiency. Incorporating transition metals with substantial antioxidative capacity into lightweight‐element‐based HEAs can greatly enhance oxidative stability, attaining elevated antioxidant activity without markedly increasing the material's density. This form of emergent behavior transcends catalysis, influencing several material attributes such as mechanical strength, magnetic performance, and thermoelectric efficiency. Given its importance, the cocktail effect is still only partially comprehended. The mechanisms regulating these synergistic interactions and the resulting structure‐property connections remain under ongoing investigation. Ongoing endeavors that integrate improved characterization methods and theoretical modeling are crucial for thoroughly understanding this intricate phenomenon and utilizing it for the systematic design of next‐generation functional materials.

## 3 Rational Design Principles for HEA Catalysts

HEAs have become prominent as advanced catalytic materials due to their remarkable compositional tunability and enhanced catalytic activity, with performance optimization significantly reliant on rational design approaches.^[^ ^45^ ^]^ The synthesis of solid‐solution HEAs is dictated by two key thermodynamic criteria: atomic radius differences between constituent elements must remain below 6.6% to minimize lattice strain, while mixing enthalpy should range between −11.6 and 3.2 kJ mol^−1^ to ensure proper miscibility, these parameters collectively determine both phase stability and catalytic activity by controlling atomic dispersion and electronic interactions.^[^ ^46^ ^]^ To further boost catalytic performance, researchers employ dual strategies of 1) structural engineering through hierarchical architectures like core‐shell structures, nanocages, and porous frameworks to maximize active surface area,^[^ ^47^ ^]^ and 2) electronic modulation via support interactions to optimize the local coordination environment and intrinsic activity of catalytic sites.^[^ ^48^ ^]^

### 3.1 Phase Structure and Morphology

**Figure 2:**
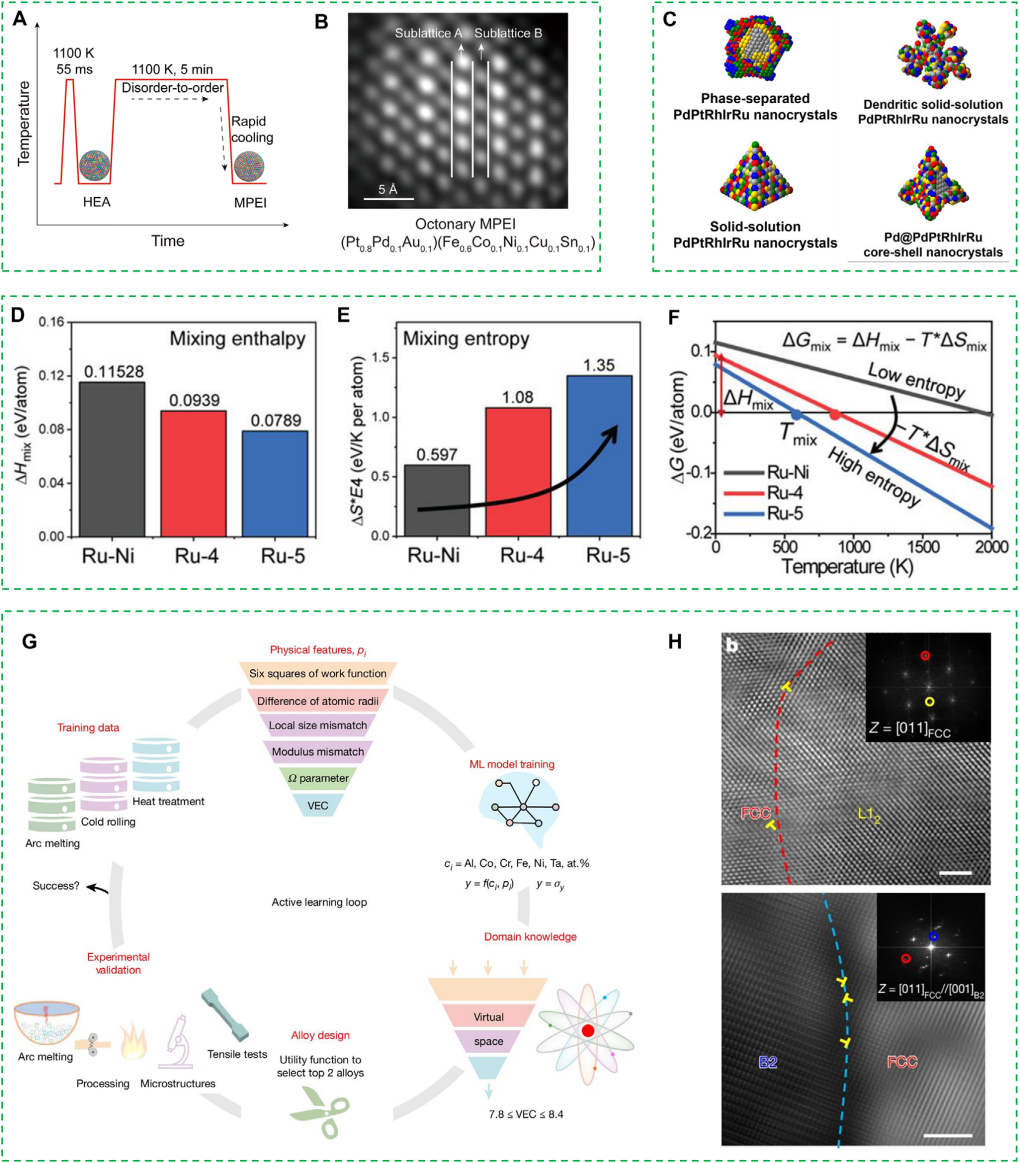
A) Temperature profile during the synthesis of HEI‐NPs. B) HAADF‐STEM image revealing the ordered L1_o_ structure in an octonary HEI‐NPs. Reproduced with permission.^[^ ^49^ ^]^ Copyright 2022, American Association for the Advancement of Science. C) Schematic representation of morphological variations in HEA nanostructures. Reproduced with permission.^[^ ^51^ ^]^ Copyright 2023, American Association for the Advancement of Science. D) Evolution of mixing enthalpy (ΔH) and E) configurational entropy (ΔS) with increasing elemental constituents. F) DFT‐predicted temperature dependence of Gibbs free energy (ΔG). Reproduced with permission.^[^ ^23^ ^]^ Copyright 2020, American Association for the Advancement of Science. G) Representing the domain knowledge‐based active learning loop consists of six steps, and H) (Top) HR‐TEM image showing the FCC/L1_2_ interfaces and the corresponding FFT pattern (inset), and (Bottom) image showing the FCC/B_2_ interfaces with the corresponding SAED (inset). Reproduced with permission.^[^ ^54^ ^]^ Copyright 2025, Springer Nature.

The catalytic activity of HEAs is strongly influenced by their crystal structure, which is inherently linked to phase composition and morphology, these structural attributes govern surface energetics, electronic configuration, and the distribution of active sites, thereby affecting reactant adsorption and reaction kinetics. Phase structure modulation can be achieved through chemical or physical approaches, with the latter primarily involving thermodynamic control via temperature, pressure, or magnetic field adjustments. Notably, post‐synthesis annealing at elevated temperatures facilitates interatomic diffusion, driving the formation of ordered high‐entropy intermetallic compounds (HEIs). Hu et al. demonstrated this principle through a disorder‐to‐order transition strategy for synthesizing HEIs NPs (**Figure** **2**A).^[^ ^49^ ^]^ Their approach involved thermally inducing atomic rearrangement in disordered solid‐solution HEA NPs precursors, followed by rapid quenching to preserve the metastable ordered configuration. The resulting L1_o_ structured HEIs NPs featured two distinct sublattices: one randomly occupied by Pt, Au, and Pd, and the other by Fe, Co, Ni, Cu, and Sn (Figure 2B). Further advancing this paradigm, Luo et al. engineered FeCoNiCuPd HEA NPs with dual‐phase characteristics through ligand‐assisted interfacial assembly coupled with NH_3_‐atmosphere annealing.^[^ ^50^ ^]^ This precise structural control yielded materials with chemically ordered phases, enhanced stability, and tailored mesoporous architectures, key attributes for optimizing catalytic performance.

The surface morphology of HEAs is principally governed by the kinetic competition between atomic deposition rates and surface diffusion rates of reactive phase constituents. This balance can be precisely modulated through three key parameters: 1) concentration gradients of reactive phase precursors, 2) controlled acceleration of reagent droplet deposition, and 3) strategic selection of solvent phase properties. Yang et al. developed a ground breaking methodology for the systematic synthesis of platinum‐group quinary HEA nanocrystals.^[^ ^51^ ^]^ Their approach combines reduction kinetic analysis with entropy optimization during nucleation, enabling precise control over metal precursor stoichiometry and injection dynamics. This paradigm facilitates the programmable fabrication of three distinct architectures, conventional solid‐solution HEAs, dendritic solid‐solution variants, and core‐shell nanostructures (Figure 2C). Building upon these principles, Yang et al. subsequently engineered a library of ten unique quinary HEA systems composed of three iron‐group metals and two platinum‐group metals.^[^ ^52^ ^]^ These systems exhibit two critical advancements, a novel square atomic packing configuration and unprecedented elemental distribution control at the atomic scale. Among these, PtRuFeCoNi HEAs demonstrated exceptional catalytic performance electrocatalytic hydrogen evolution reaction and hydrogen oxidation reaction exhibiting both superior activity and stability compared to conventional Pt/C benchmark catalysts.

### 3.2 Surface Modification

Material surfaces serve as critical interfaces for chemical reactions and physical processes, with surface engineering of HEAs offering a powerful approach to enhance their functional performance. A particularly effective strategy involves grafting tailored organic ligands onto HEA surfaces, which can impart specific functionalities such as catalytic activity, antioxidant properties, or molecular recognition capabilities. This ligand‐based modification optimizes interfacial interactions and active site exposure, enabling HEAs to perform effectively in diverse applications. A notable example of this approach was demonstrated by Liang et al.^[^ ^16^ ^]^ who developed a generalized metal‐ligand cross‐linking strategy for preparing ultrasmall PtPdRuRhIr HEAs. Their method, based on aldol condensation reactions, successfully grafted organic ligands onto the HEA surfaces. The resulting materials exhibited exceptional peroxidase‐like enzymatic activity and high photothermal conversion efficiency, showcasing how deliberate ligand functionalization can unlock multifunctional capabilities in HEAs while preserving their structural advantages.

### 3.3 Element Selection

HEAs are fundamentally governed by their chemical composition and elemental selection, where the high‐entropy mixing of multiple principal elements forms the basis for their unique characteristics. The concentration ratios and mixing states of constituent elements play a pivotal role in determining material performance, enabling property optimization through precise compositional tuning. Research has extensively explored various metallic combinations and ideal stoichiometries to achieve targeted functionalities, as the intrinsic behavior of HEAs stems directly from their elemental constitution. A notable example is the FeCoNiXRu system developed by Zhu et al. where X represents interchangeable elements (Cu, Cr, or Mn), creating diverse active sites to investigate elemental contributions.^[^ ^53^ ^]^ This system demonstrates how electronegativity differences among components drive charge redistribution, generating highly active Co and Ru sites with optimized energy barriers. Their findings establish that strategic modulation of component electronegativity can enhance catalytic performance by fine‐tuning synergistic electronic effects and optimizing energy landscapes in HEAs.

The catalytic performance of HEAs can also be systematically enhanced by precisely controlling the number of constituent elements to optimize synergistic interactions between active sites, a critical factor governing both activity and selectivity. Kitagawa et al. systematically evaluated this principle through comparative studies of ethanol oxidation reactions across progressively complex HEA systems, demonstrating measurable activity enhancements from ternary (PdPtRh) to quaternary (IrPdPtRh), pentanary (IrPdPtRhRu), and ultimately hexanary (RhRuPdOsIrPt) configurations, clearly establishing the positive correlation between elemental diversity and catalytic performance.^[^ ^55^ ^]^ While these findings underscore the importance of strategic element selection in HEA design, conventional experimental approaches for identifying optimal compositions remain constrained by substantial time and resource requirements, highlighting the need for more efficient computational or high‐throughput screening methodologies to accelerate materials discovery in this field.

### 3.4 Theoretical Designs strategy

Traditional computational approaches, such as density functional theory (DFT) and calculation of phase diagrams, have provided foundational insights into HEA behavior but are often limited by their narrow composition‐space coverage.^[^ ^56^, ^57^ ^]^ To overcome these constraints, high‐throughput screening (HTS) methodologies enabled by advanced simulations and data‐driven techniques have emerged as indispensable tools for accelerating HEA design. Recent advances in computational chemistry have further established theoretical modeling as a critical framework for guiding HEAs development, offering a more efficient alternative to conventional trial‐and‐error experimentation, which is often labor‐intensive and time‐consuming. The integration of machine learning (ML) with HTS and DFT computations has revolutionized the rapid identification of optimal elemental combinations and their potential to form single‐phase solid‐solution HEAs. Complementing these methods, molecular dynamics (MD) and Monte Carlo (MC) simulations enable precise predictions of structural evolution, phase stability, mechanical properties, and phase distributions in HEAs.^[^ ^24^, ^58^ ^]^ This synergistic paradigm, combining multiscale computational predictions with experimental validation, significantly enhances the precision and efficiency of HEAs discovery and optimization.

Yao et al. systematically investigated the thermodynamic behavior of RuNi, RuRhCoNi, and RuRhCoNiIr alloys by evaluating their mixing enthalpy (ΔH_mix_), mixing entropy (ΔS_mix_), and Gibbs free energy (ΔG) as functions of temperature.^[^ ^23^ ^]^ Their analysis revealed that increasing the number of constituent elements leads to a progressive decline in (ΔH_mix_) and a pronounced enhancement in (ΔS_mix_) (Figure 2D,E). Notably, ΔG exhibited a linear dependence on temperature, decreasing monotonically and transitioning to negative values beyond a critical threshold (Figure 2F). This trend underscores the stabilizing role of elevated temperatures, while the inclusion of additional elements lowers the requisite temperature for solid‐solution formation. To further elucidate kinetic effects, the authors employed MD simulations to analyze Ru diffusion in Ru_0.25_Rh_0.25_Co_0.2_Ni_0.2_Ir_0.1_ HEA NPs. The self‐diffusion coefficient of Ru in this multicomponent system was found to be approximately two orders of magnitude lower than in the binary Ru_0.25_Ni_0.25_ alloy. This suppression of atomic mobility arises from severe lattice distortion induced by homogeneous elemental mixing, a phenomenon central to the sluggish diffusion effect that enhances HEA stability. In a parallel study, Cavin et al.^[^ ^59^ ^]^ pioneered the concept of 2D high‐entropy transition metal dichalcogenides incorporating group V and VI transition metals. Using DFT, they quantified the stability of these alloys by evaluating formation enthalpy and configurational entropy changes during synthesis. The Gibbs free energy profiles of stable phase mixtures across a temperature range enabled estimation of the growth temperature window for HEAs above their decomposition thresholds. Guided by these computational predictions, the authors successfully synthesized three high‐entropy TMDCs (MoWNbTa)S, (MoWNbV)S, and (MoWVNbTa)S, demonstrating the efficacy of theory‐driven material design.

Recent work by Rao et al. demonstrates the effectiveness of ML in accelerating HEA discovery through an active learning approach combined with a two‐stage ensemble regression framework.^[^ ^60^ ^]^ Their method establishes target properties, evaluates physical descriptors, and iteratively refines predictions via experimental feedback. Notably, models incorporating physical descriptors outperformed composition only approaches in predicting thermal expansion behavior. The team developed a closed‐loop system featuring i) generative autoencoders for composition design, ii) a hybrid neural network/boosting tree evaluator, and iii) experimental validation to optimize predictions. This ML‐DFT experimental framework successfully identified high‐performance Invar alloys while highlighting the importance of physics‐informed descriptors for accurate property prediction, offering both accelerated discovery and deeper insights into composition‐property relationships in HEAs. Building on these advances, Sohail et al. have pushed the boundaries of ML‐driven HEA design by integrating domain‐specific knowledge with active learning to achieve unprecedented mechanical performance.^[^ ^54^ ^]^ Their framework (Figure 2G) employed physical descriptors, including atomic radius mismatch and mixing enthalpy to constrain the compositional search space, enabling iterative ML‐guided experiments that culminated in the discovery of Fe_35_Ni_29_Co_21_Al_12_Ta_3_. This optimized composition facilitated dual‐phase precipitation, with microstructural validation via TEM (Figure 2H) revealing 66.6 vol% coherent L1_2_ nanoprecipitates for strengthening and 15 vol% deformable B_2_ micro precipitates that sustained strain hardening, collectively enabling a record 1.8 GPa yield strength with 25% uniform elongation. By coupling ML predictions with targeted processing (e.g., aging at 750 °C), they achieved a microstructure unattainable through traditional methods, setting a new benchmark for strength‐ductility synergy in HEAs.

## 4 Synthesis Strategies

A significant advancement has been made in the synthesis of HEAs, with methodologies broadly classified into solid‐state, liquid‐phase, and gas‐phase routes.^[^ ^61^ ^]^ Mechanical alloying remains the most widely adopted solid‐state technique, offering scalability and cost‐effectiveness for bulk HEA production. However, this method faces limitations such as high energy input, potential oxidation during processing, and inhomogeneous mixing, often necessitating post‐treatment via compaction and sintering to achieve desired microstructural uniformity.^[^ ^62^ ^]^ Liquid‐state methods, including arc melting and laser‐assisted techniques, enable effective elemental mixing but require elevated temperatures to overcome kinetic barriers and ensure complete homogenization. A common challenge is the formation of dendritic or segregated microstructures, which typically require prolonged thermal annealing to achieve equilibrium phase distribution.^[^ ^63^, ^64^ ^]^ Vapor‐phase techniques such as magnetron sputtering, atomic layer deposition, and chemical vapor deposition provide exceptional control over composition and microstructure at the nanoscale. While these methods enable the synthesis of high‐purity HEAs with tailored properties, they often involve complex instrumentation, high operational costs, and slower deposition rates compared to bulk synthesis routes.^[^ ^65^ ^]^ Each approach presents distinct trade‐offs between scalability, energy efficiency, microstructural control, and economic viability, necessitating careful selection based on target alloy properties and intended applications.

Recent advances in HEAs research have focused on developing innovative synthesis strategies with particular emphasis on overcoming the challenges associated with nanoscale HEA fabrication. The synthesis of nanoscale HEAs presents unique thermodynamic and kinetic hurdles, including maintaining single‐phase stability during co‐reduction of multiple metallic precursors, preventing elemental segregation due to differing reduction potentials, and controlling nucleation/growth processes to ensure homogeneous elemental distribution.^[^ ^66^ ^]^ These challenges underscore the critical need for fundamental investigations into nanoscale growth mechanisms to enable precise control over phase purity, particle size, and compositional uniformity. Current research efforts are increasingly directed toward developing tailored synthesis methods that can address these limitations while meeting application‐specific requirements for microstructure and properties. The following discussion will comprehensively examine contemporary nanoscale synthesis techniques, analyzing their effectiveness in producing well‐defined HEA nanostructures with controlled characteristics.

### 4.1 Carbon‐Thermal Shock (CTS) Approach

**Figure 3:**
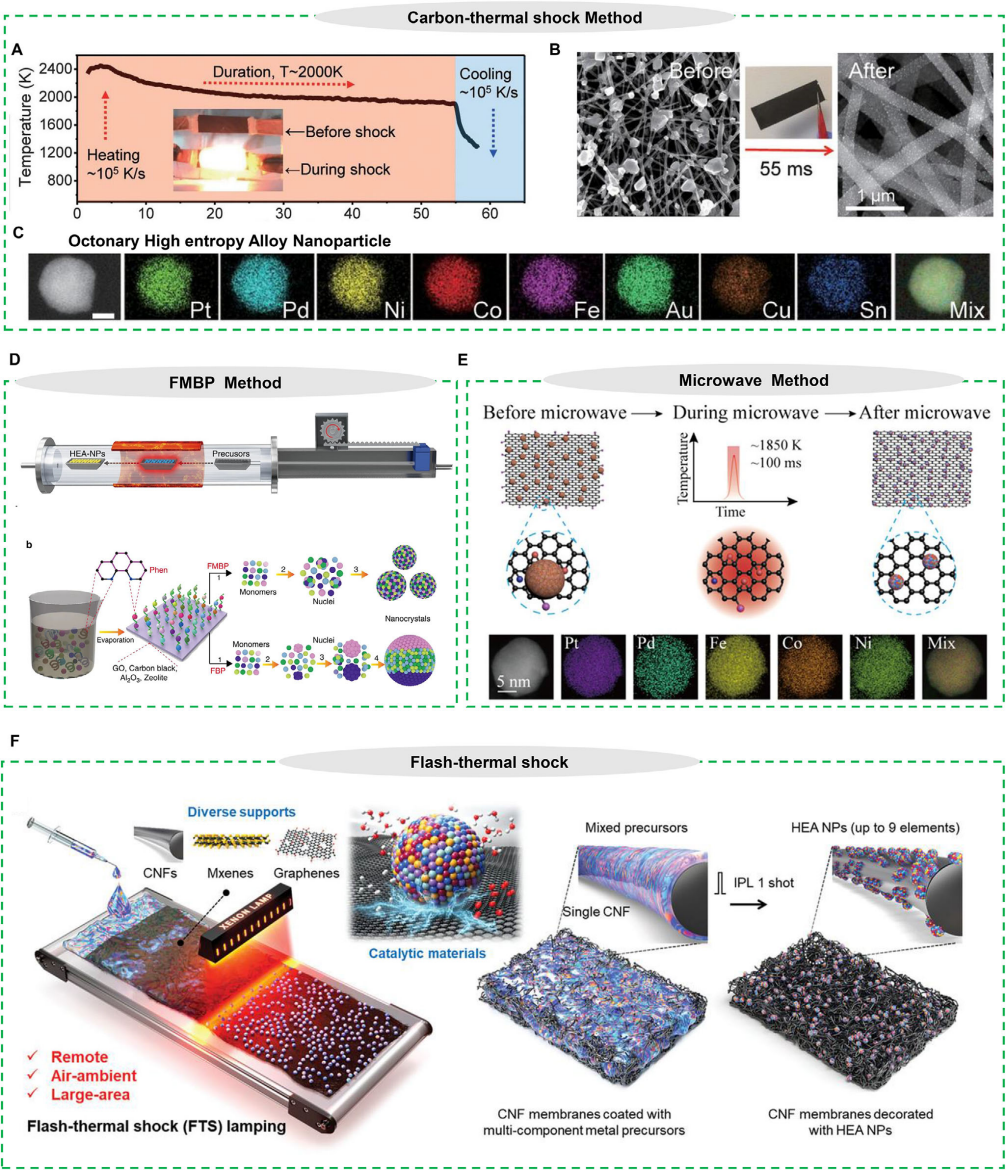
A) Temperature profile during 55‐ms thermal shock processing. B) SEM images showing precursor salts on CNF supports (left) and resulting (PtNi) nanoparticles after CTS (right). C) EDS mapping of the eight components in HEA NPs. Reproduced with permission.^[^ ^67^ ^]^ Copyright 2018, American Association for the Advancement of Science. D) Fast‐moving bed pyrolysis illustration. Reproduced with permission.^[^ ^68^ ^]^ Copyright 2020, Springer Nature. E) Microwave heating mechanism illustration. Reproduced with permission.^[^ ^71^ ^]^ Copyright 2021, American Chemical Society. F) Schematic representation of the non‐contact, mass‐production synthesis of HEA NPs under ambient conditions using millisecond‐scale (<20 ms) pulsed light irradiation from a xenon lamp. Reproduced with permission.^[^ ^72^ ^]^ Copyright 2023, Wiley‐VCH GmbH.

The carbon‐thermal shock (CTS) approach, initially presented by Hu et al. signifies a significant advancement in the synthesis of metallic alloys and nanostructured materials with enhanced characteristics.^[^ ^67^ ^]^ This technique utilizes exceptionally high temperatures (≈2000K) and rapid cooling (≈10^5^ K.s^−1^) to facilitate the swift synthesis NPs on substrates (**Figure** **3**A). As a type of vapor‐phase deposition, this technique can produce nanostructured materials with exceptional homogeneity, achieving both uniform elemental dispersion and precise compositional control at the atomic scale. Hu et al. established the adaptability of this method by effectively manufacturing HEA NPs composed of up to eight unique metallic elements, namely Pt, Pd, Ni, Co, Fe, Au, Cu, and Sn. The swift temperature increase during CTS causes the instantaneous breakdown of metal precursors and the spontaneous amalgamation of the resultant metal species, facilitating effective mixing and alloying at the nanoscale (Figure 3B). Through this strategy, a broad range of multicomponent HEA NPs was successfully developed, exhibiting consistent elemental distribution (Figure 3C). Notwithstanding its efficacy, the existing CTS approach depends on carbon‐based substrates and is constrained by comparatively poor manufacturing yields. Improving the scalability and productivity of CTS is crucial for advancing its use in industrial scale HEA catalyst production. Ongoing study and optimization are required to enhance this technology for the large‐scale synthesis of HEAs with accurately regulated composition, dimensions, and morphology.

### 4.2 Fast‐Moving Bed Pyrolysis (FMBP)

The fast‐moving bed pyrolysis (FMBP) method has recently demonstrated exceptional capability for fabricating ultrasmall, well‐dispersed HEA nanoparticles on diverse supports such as carbonaceous materials, zeolites, and γ‐Al_2_O_3_ (Figure 3D). This development overcomes a significant constraint of the CTS method, which predominantly functions well with carbon‐based supports. The FMBP process consists of three distinct stages: The process of converting precursors into monomers and then transforming the monomers into nuclei, and the growth of nuclei into nanocrystals. By strategically modulating reaction temperature and nucleation kinetics, the nucleation barrier and thermodynamic stability of the system can be precisely tuned, enabling controlled formation of HEA NPs with tailored composition and size distributions. The FMBP technique facilitates the creation of structurally uniform HEAs, which are distinguished by their minimal free energy combined with fast nucleation behavior. As an example, Gao et al.^[^ ^68^ ^]^ demonstrated the successful application of FMBP to deposit uniform 2 nm HEA NPs on granular supports, even under high‐temperature conditions. The rapid thermal processing enables the development of small nuclei clusters and consistent HEA NPs, while also efficiently inhibiting phase separation. The FMBP method demonstrates considerable potential for generating ultrasmall, highly crystalline multicomponent HEAs, ensuring a uniform distribution across different support materials.

### 4.3 Microwave Synthesis

Microwave‐assisted synthesis has gained prominence in metallurgical applications due to its unique benefits, including targeted heating, bulk energy deposition, and enhanced sintering performance.^[^ ^69^, ^70^ ^]^ This process has recently attracted significant interest for synthesizing HEAs, offering a feasible alternative to traditional melting techniques. Unlike conventional thermal methods, microwave‐assisted synthesis relies on direct dielectric coupling between electromagnetic energy and the target material, subsequently transforming it into thermal energy. This transpires via the fast oscillation of dipolar molecules within the substance, producing “internal friction heat” that elevates the temperature. Building upon this foundation, Hu et al. developed an advanced microwave synthesis strategy utilizing graphene oxide as a microwave‐absorbing substrate, generating ultra‐rapid temperature spikes reaching 1850 K within seconds (Figure 3E).^[^ ^71^ ^]^ This extreme heating rate facilitated the successful synthesis of PtPdFeCoNi HEA NPs, where simultaneous decomposition of precursor elements at these elevated temperatures promoted immediate liquefaction and homogeneous mixing by passing traditional diffusion limitations. The resulting HEA NPs exhibited uniform composition and controlled particle sizes ≈12 nm. Compared to conventional furnace‐based methods, microwave synthesis offers distinct advantages, including instantaneous, volumetric heating and exceptional compatibility with carbon‐based materials across various dimensional scales, establishing it as a promising platform for next‐generation HEA fabrication.

### 4.4 Flash‐Thermal Shock (FTS)

Despite the advantages of the CTS method, its practical application faces several limitations. First, CTS is inherently restricted to conductive materials, as it requires direct electrode contact with carbon‐based supports to facilitate sufficient current flow for Joule heating, a process typically conducted under vacuum conditions. Furthermore, the effectiveness of Joule heating is strongly influenced by the consistency and homogeneity of the carbon substrates, which can result in non‐uniform heat distribution when processing larger sample areas. These constraints highlight the need for an alternative synthesis approach that overcomes these challenges while maintaining compatibility with ambient atmospheric conditions, scalability for large‐area processing, and versatility across a broader range of materials without stringent conductivity requirements. Recently, Cha et al.^[^ ^72^ ^]^ developed a breakthrough photothermal synthesis technique that overcomes several limitations (Figure 3F). This approach demonstrates three key advantages: 1) ambient environment compatibility, 2) scalable large‐area processing capability, and 3) material‐agnostic operation. The method utilizes pulsed light irradiation (4.9 J cm^−2^, 20 ms duration) on photon‐absorbing substrates like carbon nanofibers, graphene oxide, and MXenes to generate instantaneous high temperatures. This flash heating enabled the successful synthesis of nine‐element HEAs through rapid thermal processing. The non‐contact nature of this photothermal mechanism represents a significant advancement in HEAs synthesis, as it eliminates the need for direct material electrode contact, avoids complex vacuum system requirements, and allows for precise, localized heating control. Moreover, its compatibility with flexible substrates and operation under atmospheric conditions highlights its potential for scalable, industrial‐level production.

### 4.5 Laser‐Scanning Ablation Technology (LSA)

**Figure 4:**
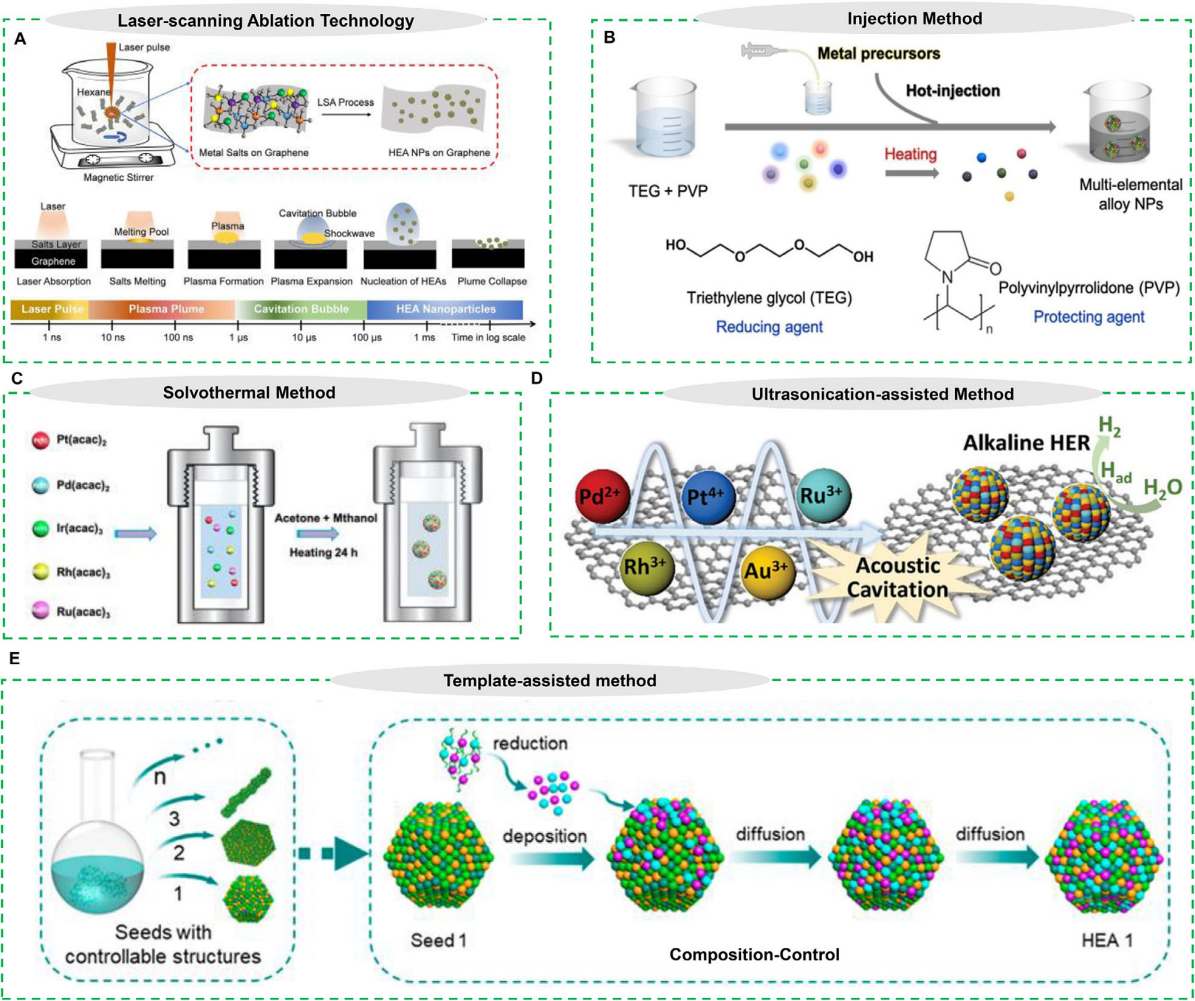
A) Schematic illustration of the LSA strategy and different events occurring on the graphene surface after the laser pulse, with time scale. Reproduced with permission.^[^ ^75^ ^]^ Copyright 2023, Elsevier. B) Representation of the Injection method. Reproduced with permission.^[^ ^65^ ^]^ Copyright 2022, Wiley‐VCH GmbH. C) Protocol for the synthesis of PtPdIrRhRu nanoparticles via the solvothermal method. Reproduced with permission.^[^ ^82^ ^]^ Copyright 2019, Wiley‐VCH GmbH. D) schematic illustration of synthesis of HEA‐NPs/carbon (PtAuPdRhRu supported on XC‐72 carbon) catalysts, HAADF and EDS elemental maps of the prepared NPs. Reproduced with permission.^[^ ^83^ ^]^ Copyright 2019, Wiley‐VCH GmbH. E) Schematic illustration of template‐assisted synthesis. Reproduced with permission.^[^ ^84^ ^]^ Copyright 2023 American Chemical Society.

This technique has emerged as a viable method for generating high‐quality, repeatable, and compositionally controllable HEA NPs, with extensive potential for diverse applications.^[^ ^73^, ^74^ ^]^ This method involves directing a high‐energy laser beam onto a bulk HEA target, generating a plasma plume through rapid vaporization. The plume comprises hot atoms, ions, and atomic clusters that condense upon cooling to produce HEA NPs. The nanoparticles are subsequently cooled swiftly and gathered in a liquid media, such as ethanol, generally resulting in particles with an average diameter of under 5 nm. Zou et al.^[^ ^75^ ^]^ recently employed a straightforward laser scanning ablation (LSA) technique to produce PtIrCuNiCr HEAs (**Figure** **4**A). Metal salts with specified atomic percentages were deposited on a graphene substrate and irradiated by a laser. The technique produced a highly compressed plasma with several metal species, enabling the fast synthesis of HEA nanoparticles. This LSA approach is exceptionally versatile, facilitating the fast generation of HEAs across a range of alloy compositions, specific precursors, and substrate materials, rendering it particularly advantageous for high‐throughput nanomaterial screening.

### 4.6 Wet Chemical Approach

The fabrication of HEAs by wet chemical or solution‐based synthesis highlighting unique capabilities in materials research. This method facilitates the integration of several metal precursors under comparatively moderate circumstances, providing a flexible alternative to conventional high‐temperature synthesis approaches.^[^ ^71^ ^]^ The primary benefit of wet chemical synthesis is its capacity to precisely regulate the formation of HEA NPs by manipulating essential parameters, including reaction temperature, selection of reducing agents, surfactants, ligands, and reaction time. The subsequent section will comprehensively examine various representative wet chemical synthesis techniques, highlighting their unique characteristics and contributions to the progression of HEA nanomaterial development.

#### 4.6.1 Colloidal Synthesis

Solution‐phase colloidal synthesis enables precise fabrication of uniform nanoparticles with tailored composition, morphology, and dimensions, making it particularly suitable for developing complex multi‐metallic alloy nanostructures.^[^ ^76^, ^77^ ^]^ Metal precursor reduction typically occurs in organic media (e.g., oleylamine or 1‐octadecene) with surfactant stabilization, conducted under controlled thermal conditions (150–350 °C range).^[^ ^78^ ^]^ Surface ligands are essential in modulating nanoparticle morphology and size by inhibiting aggregation and stabilizing development via the establishment of ionic or covalent interactions on the nanoparticle surface.^[^ ^79^ ^]^ Colloidal synthesis methods are primarily categorized into two types: the one‐pot approach and the hot‐injection method, each providing unique benefits in regulating nucleation and growth kinetics.

The one‐pot solution‐phase method, characterized by the concurrent introduction of various metallic precursors into a unified reaction mixture, is extensively utilized frequently alongside other methodologies to enhance the structural or compositional attributes of HEAs. For example, Li et al.^[^ ^80^ ^]^ adeptly synthesized uniformly spherical Pt_18_Ni_26_Fe_15_Co_14_Cu_27_ NPs, exhibiting an average size of 3.4 ± 0.6 nm through this uncomplicated methodology. A prevalent approach involves the calcination‐assisted technique, which improves the nano/microstructure of HEAs by facilitating atomic diffusion and the reconstruction of nanostructures during the annealing process following synthesis. The microstructural attributes of HEA NPs exhibit considerable sensitivity to variations in annealing temperature, duration, and atmospheric conditions. Notably, single‐phase NiCoFePtRh NPs with a record‐small average diameter of 1.68 nm (the smallest reported for HEAs) were fabricated through NaBH_4_ reduction of mixed metal precursors, succeeded by H_2_/Ar annealing at 350 °C.^[^ ^81^ ^]^ The results highlight the significant opportunity to refine HEA microstructures by meticulously adjusting annealing parameters.

Injection‐based synthesis provides fine regulation of nanoparticle size and chemical composition, and dispersity by modifying factors such as injection rate, temperature, and precursor concentration. Two prevalent versions of this methodology are the hot‐injection and slow‐injection techniques. During the hot‐injection process, metal precursor solutions are rapidly introduced into a preheated mixture containing a reducing agent (e.g., triethylene glycol) and a stabilizer (e.g., polyvinylpyrrolidone, PVP) (Figure 4B).^[^ ^65^ ^]^ This fast injection facilitates the concurrent reduction of all metal ions, promoting homogeneous nucleation and the formation of alloy NPs.

The slow‐injection approach entails the gradual introduction of the precursor solution to reduce the probability of atomic collisions among identical elements. This facilitates the inhibition of isolated monometallic nanoparticle formation and promotes uniform alloy development. Dey et al.^[^ ^85^ ^]^ successfully produced multiple HEA NPs such as NiPdPtRhIr, NiFeCoPdPt, SnPdPtRhIr, and NiSnPdPtIr using the slow‐injection method. Initially, individual metal salts were dissolved in a minimal quantity of oleylamine and agitated under vacuum for 15 min to achieve thorough degassing. A mixture of octadecene and oleylamine was heated and stirred at 120 °C for 30 min under an argon atmosphere to create an inert environment. The pre‐prepared metal salt solutions were there after injected into the heated reaction mixture at 275 °C (315 °C for NiFeCoPdPt) at a regulated rate of 0.4 mL min^−1^ over a duration of 10 min. Following the process, homogenous alloy nanoparticles exhibiting spherical morphology and well‐defined crystal lattices, ≈10 nm in diameter, were produced. The results underscore the efficacy of the slow‐injection technique in facilitating homogenous alloy production.

#### 4.6.2 Solvothermal Synthesis

Solvothermal synthesis using solvents such as oleylamine or polyols has proven to be an efficient and economical approach for synthesizing HEAs, enabling the incorporation of multiple metallic elements. Bondesgaard et al.^[^ ^82^ ^]^ developed a low‐temperature solvothermal method to fabricate PtPdIrRhRu HEA NPs. The process involved dissolving metal precursors, primarily acetylacetonate complexes, in a mixture of acetone and ethanol, followed by transferring the solution into a Teflon‐lined stainless‐steel autoclave (Figure 4C). The reaction proceeded at 200 °C for 4–24 h, resulting in the formation of PtPdIrRhRu HEA NPs. The strong coordination between metal ions and acetylacetonate ligands was found to delay precipitation, thereby facilitating the simultaneous co‐precipitation of multiple metal species. Additionally, the crystallite size of the synthesized HEA nanoparticles was influenced by the enthalpy of fusion (or melting point) of the constituent metals, suggesting that these thermodynamic properties could serve as useful indicators for predicting nanoparticle size in alloys with varying stoichiometric compositions

#### 4.6.3 Ultrasonication‐Assisted Wet Chemistry Synthesis

Ultrasonication‐assisted synthesis provides a rapid and versatile approach for fabricating HEAs on diverse substrates, often at room temperature and pressure. As reported by Liu et al.^[^ ^83^ ^]^ this technique successfully generated PtAuPdRhRu nanoparticles averaging ≈3 nm in size (Figure 4D). The method relies on acoustic cavitation effects, which create transient microscopic hot spots with extreme temperatures (approaching 5000 °C) and pressures (exceeding 200 MPa) that persist for nanosecond durations. These intense localized conditions facilitate the nearly instantaneous co‐reduction of metal precursors in ethylene glycol, serving dual roles as both reaction medium and reducing agent, allowing alloy nanoparticle formation within minutes. While the approach faces challenges regarding reaction kinetics and product yield, the synthesized HEAs consistently demonstrate excellent crystalline quality, low defect concentrations, and straightforward processability. Like solvothermal approaches, ultrasonication enables precise control over HEA nanomaterial characteristics, including morphology and phase composition in solution‐phase reactions.

#### 4.6.4 Template‐Assisted Method

The ability to fabricate nanoscale HEAs with precisely controlled compositions and customized morphologies represents a critical advancement for developing next‐generation catalytic materials. As reported, Huang et al.^[^ ^84^ ^]^ developed an innovative seed‐mediated growth approach that uses pre‐synthesized nanocrystals as templates to direct the formation of HEA nanostructures. This technique achieves exceptional compositional control through a carefully orchestrated process involving three key steps: 1) controlled reduction of metal precursors, 2) surface deposition of metal atoms, and 3) thermally activated atomic interdiffusion. Using this strategy, the research team successfully fabricated an extensive library of HEA nanostructures with varying dimensionalities, including 0D nanoparticles, 1D nanowires, 2D ultrathin nano rings, and 3D dendritic architectures. These structures incorporated five or more metallic elements from a diverse selection (Figure 4E). The synthesis mechanism initiates with nano seed formation, which subsequently acts as a structural scaffold. When metal precursors are introduced, the liberated atoms first deposit onto the seed surface before undergoing inward diffusion along chemical potential gradients, ultimately forming homogeneous alloyed structures. This approach offers remarkable versatility in HEA design, as the final architecture can be precisely tailored by engineering the initial seed characteristics.

### 4.7 Electrochemical Deposition Synthesis

**Figure 5:**
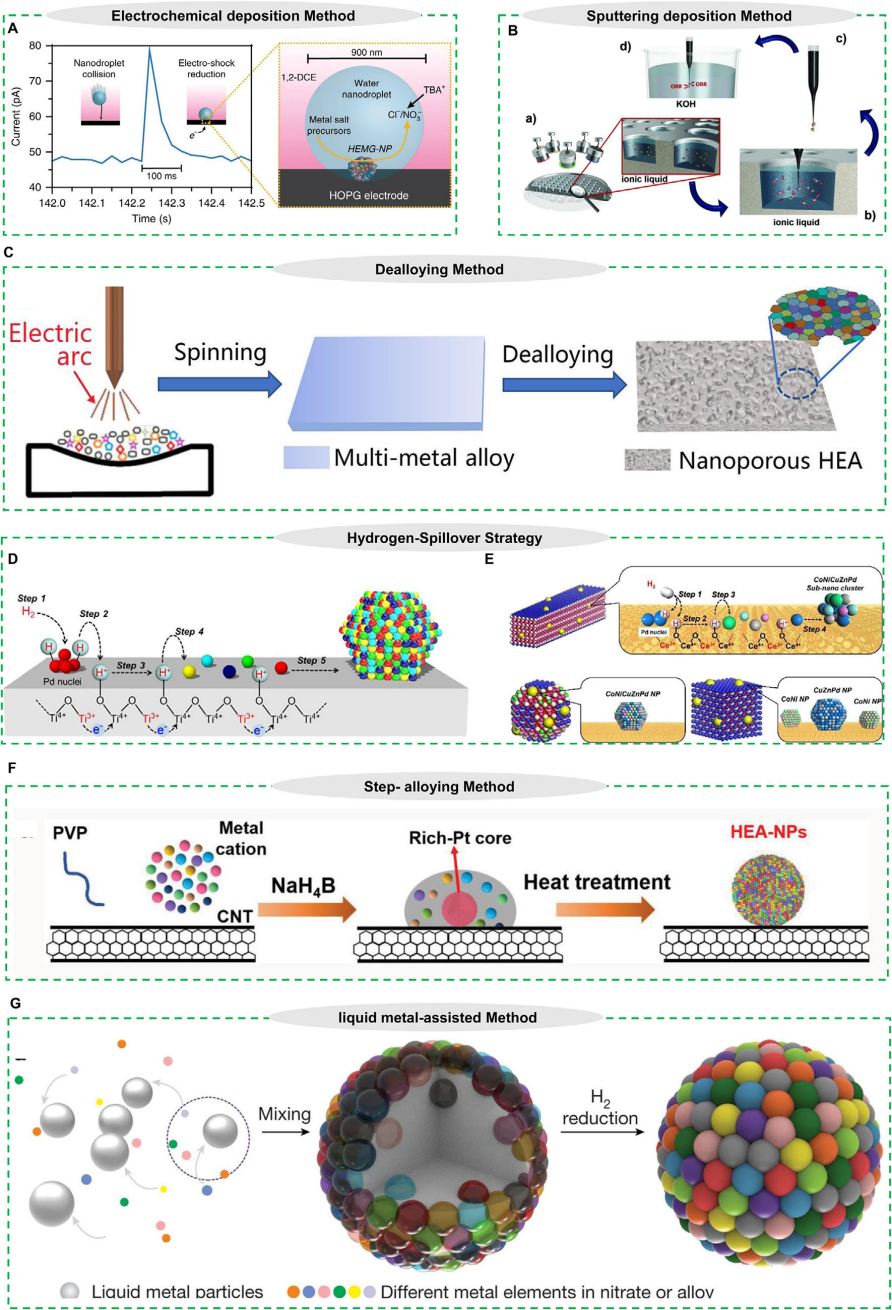
A) Transient current during the collision of a single nanodroplet on a carbon fiber in nanodroplet‐mediated electrodeposition. Reproduced with permission.^[^ ^86^ ^]^ Copyright 2019, Springer Nature. B) Ionic liquid combinatorial co‐sputtering for NPs synthesis. Reproduced with permission.^[^ ^92^ ^]^ Copyright 2018, Wiley‐VCH GmbH. C) Schematic illustration of the preparation of binder‐free nanoporous. Reproduced with permission.^[^ ^95^ ^]^ Copyright 2022, American Chemical Society. D) Schematic illustration of the elementary steps of the formation mechanism of HEA NPs. Reproduced with permission.^[^ ^96^ ^]^ Copyright 2021, Springer Nature. E) Schematic illustration of elementary steps of the formation mechanism of HEA clusters on CeO_2_‐NRs, CeO_2_‐NPs, and CeO_2_‐NCs, respectively. Reproduced with permission.^[^ ^97^ ^]^ Copyright 2023, American Chemical Society. F) Schematic diagram of step alloying strategy for synthesis of HEA NPs. Reproduced with permission.^[^ ^98^ ^]^ Copyright 2023, Wiley‐VCH GmbH. G) Schematic diagram of the liquid metal‐assisted synthesis process. Reproduced with permission.^[^ ^26^ ^]^ Copyright 2023, Springer Nature.

Electrodeposition serves as a versatile and reliable technique for synthesizing metallic nanoparticles with precise control over nucleation and growth kinetics.^[^ ^86^ ^]^ By applying an external electric field, this method drives redox reactions at the electrode‐electrolyte interface within an electrochemical cell typically composed of a metal oxide cathode, graphite anode, and either molten salt or organic electrolyte.^[^ ^87^ ^]^ The deposition process and final material characteristics are strongly influenced by several key parameters, including the metal oxide precursor type, applied potential, operating temperature, and electrolysis duration. Tong et al. pioneered the direct electrodeposition of HEAs, successfully fabricating BiFeCoNiMn HEA nanorods using a mixed organic electrolyte system. Subsequent analysis revealed that the resulting HEA coatings exhibited an amorphous structural arrangement.^[^ ^88^ ^]^ Recently, Dick et al.^[^ ^86^ ^]^ presented an innovative technique termed transient electrodeposition, which facilitates the creation of high‐entropy metallic glasses by the impact of nanodroplets on electrode surfaces (**Figure** **5**A). This method involves encapsulating metal precursor salts within water nanodroplets, which are subsequently emulsified in dichloroethane. Upon contact with the electrode, the confined metal precursors experience co‐electrodeposition within the droplets. The ultrafast response rates, ≈100 ms, prevent the metal atoms from organizing into a crystalline lattice, leading to an amorphous, glassy form. This rapid, targeted synthesis technique creates new opportunities for producing compositionally intricate, non‐crystalline HEA materials.

### 4.8 Sputtering Deposition

This is a widely used technique for producing thin films and nanoparticles on a variety of substrates.^[^ ^89^, ^90^ ^]^ The process involves bombarding a target material with energetic ions in a vacuum environment, causing atomic ejection and subsequent condensation onto substrates to form uniform coatings. Unlike electrospinning, a distinct method where electric fields transform polymer solutions into solid fibers, sputtering enables precise control over deposition parameters. Recent advances include successful magnetron sputtering of FeMnCoCrC HEA coatings on high‐speed steel, where substrate bias voltage critically determines coating characteristics.^[^ ^91^ ^]^ Schuhmann et al.^[^ ^92^ ^]^ developed an innovative variation where argon plasma bombardment ejects target atoms into ionic liquids rather than onto solid substrates, enabling controlled nucleation and growth of CrMnFeCoNi HEA NPs with tunable sizes (Figure 5B). While offering excellent control over nanostructure morphology and composition, sputtering requires specialized vacuum equipment, potentially increasing costs.

### 4.9 Dealloying Synthesis

The dealloying technique has proven particularly effective for creating HEA NPs with enhanced specific surface areas, building on its established success in producing nanoporous metallic architectures.^[^ ^93^ ^]^ This approach works by selectively corroding the more reactive components from multicomponent solid solutions, leaving behind a porous network of the relatively noble elements. The standard procedure involves three key stages: alloy melting, rapid cooling, and the dealloying process itself. Qiu et al.^[^ ^94^ ^]^ demonstrated the effectiveness of this method by synthesizing several HEA systems, including AlNiCuPtPdAu and its derivatives with added Co/Fe. Their protocol began with preparing alloy ribbons through melt‐spinning of precisely weighed high‐purity metals (>99.9 wt.%) in an argon‐protected induction furnace. Subsequent chemical dealloying in 0.5 M NaOH solution yielded HEA NPs exhibiting a distinctive bimodal pore structure (5–8 nm pore sizes). Building on these findings, the Qiu research group developed an advanced two‐step synthesis route capable of producing single‐phase nanoporous HEAs with exceptional elemental homogeneity (Figure 5C). Their method successfully incorporated either 12 to 16 elements through a streamlined single‐step dealloying process in (NH_4_)_2_SO_4_ solution.^[^ ^95^ ^]^ While this technique offers advantages in production yield and scalability for industrial applications, its requirement for high‐temperature processing under inert atmospheres presents cost challenges.

### 4.10 Other Synthesis Methods

#### 4.10.1 Hydrogen‐Spillover Strategy

A promising strategy for the low‐temperature synthesis of supported HEA NPs involves leveraging the hydrogen spillover effect on reducible metal oxides. As demonstrated by Mori et al.^[^ ^96^ ^]^ this approach enables the fabrication of quinary CoNiCuRuPd HEA NPs on TiO_2_ at temperatures as low as 400 °C via a simple impregnation and reduction process (Figure 5D). The mechanism is driven by pronounced hydrogen spillover, facilitated by coupled proton/electron transfer on the reducible support. Initially, Pd^2+^ precursors are reduced to form nuclei that dissociate H_2_ into active hydrogen atoms. These spilled‐over hydrogen species migrate rapidly across the support surface, simultaneously reducing neighboring metal cations (Co, Ni, Cu, Ru) and promoting the formation of homogeneous, nanoscale HEA particles.

Building on this concept, Hashimoto et al.^[^ ^97^ ^]^ recently extended the hydrogen spillover‐driven synthesis to sub‐nanometric HEA clusters (<1 nm) by utilizing morphology‐controlled CeO_2_ supports (Figure 5E). They demonstrated that CeO_2_ nanorods, which preferentially expose highly reducible (110) facets, exhibit a significantly enhanced hydrogen spillover effect compared to CeO_2_ nanocubes or NPs. This powerful spillover facilitates the simultaneous reduction of a five‐component system (Co, Ni, Cu, Zn, Pd) with vastly different reduction potentials, leading to the formation of ultra‐small, well‐mixed HEA clusters at low temperatures. This method stands in contrast to non‐reducible supports like Al_2_O_3_, which lack efficient hydrogen migration pathways and result in larger, segregated particles. The resulting sub‐nanometric HEA clusters exhibit unique quantum size effects and exceptional catalytic performance, attributed to synergistic cocktail effects and a novel structural reversibility under redox cycles, where non‐noble metals act as sacrificial agents to protect noble metals from oxidation. The hydrogen spillover‐driven synthesis not only offers a facile and scalable route to supported HEA catalysts but also opens new avenues for designing advanced multifunctional materials with tailored compositions.

#### 4.10.2 Step‐Alloying Methods

The step‐alloying strategy is a two‐step synthesis approach that initially forms a core‐rich phase through a liquid phase reaction, followed by the controlled diffusion of additional components into the core during thermal treatment. Recently, Li et al.^[^ ^98^ ^]^ successfully fabricated a series of homogeneous HEAs NPs containing up to 14 elements, including strongly immiscible combinations, using this method (Figure 5F). In the first step, elements with higher solution‐phase electronegativity (e.g., Pt) are reduced by NaBH_4_, forming ultrasmall Pt‐rich cores. Simultaneously, unreduced elements and PVP co‐precipitate into amorphous nanosheets that encapsulate the metal‐rich cores. During the subsequent heat treatment at a relatively low temperature (973 K), PVP decomposes, and the remaining unreduced elements gradually diffuse into the Pt‐rich cores. Notably, the mixing enthalpy (ΔH_mix_) of Pt with other elements remains below the phase‐separation threshold, confirming that the formation of Pt‐rich cores enables the incorporation of typically immiscible elements into a single‐phase HEA at lower temperatures. This method offers a scalable and energy‐efficient route to synthesize complex HEAs with homogeneous elemental distribution, even for traditionally incompatible metal combinations.

#### 4.10.3 Liquid Metal Assisted Method

The liquid metal‐assisted synthesis strategy is a low‐temperature approach that utilizes liquid metal as a reactive medium to achieve homogeneous mixing of multiple metallic elements. Recently, Fu et al.^[^ ^26^ ^]^ has successfully fabricated complex HEA NPs containing up to 9 elements, including normally immiscible combinations, using this method (Figure 5G). In the synthesis process, metal precursors are first dispersed within nanoscale liquid Ga droplets at room temperature. During thermal treatment at 923K, the precursors undergo simultaneous decomposition and reduction, while the liquid Ga matrix facilitates atomic diffusion and mixing. The system is then cooled at controlled rates to preserve the high‐entropy state. Notably, Ga negative mixing enthalpy with other metals helps overcome thermodynamic barriers, while its liquid state provides a dynamic mixing environment that enables alloy formation at significantly lower temperatures than conventional methods. This dual effect of enthalpy reduction and enhanced atomic mobility allows for the incorporation of typically incompatible elements without phase separation. This liquid metal‐mediated approach provides a versatile and scalable route to synthesize multicomponent HEA NPs under mild conditions.

In summary, the choice of an appropriate synthesis method for HEA catalysts depends on multiple factors, including equipment requirements, operating conditions, production cost, particle size control, surface area, elemental flexibility, and overall productivity. As outlined in **Table** **2**, each approach offers distinct advantages but also presents certain trade‐offs. For practical and industrial applications, future efforts should prioritize strategies that combine high productivity with low cost and precise compositional control. Although significant progress has been made, developing scalable, economical, and versatile synthesis routes remains a major challenge. Continued innovation in this area will be crucial for unlocking the full catalytic and energy‐conversion potential of HEAs, and will require close integration of materials science, chemistry, and engineering expertise.

**Table 2:** Summary of different synthesis methods for HEAs.

Synthetic method	Temperature and pressure	Productivity	Size	Element applicability	Refs.
Carbon‐thermal shock synthesis	≈2000K/1atm	≈100mg	3–25nm	Pt, Pd, Au, Ru, Rh, Fe, Co, Ni, Cu, Mo, Sn, Ce	[67]
Fast‐moving bed pyrolysis	≈923K/1atm	≈100mg	2–50nm	Rh, Pd, Ir, Pt, Au, Mn, Co, Ni, Cu, Sn	[68]
Micro wave assisted synthesis	≈1050K/1atm	≈100mg	10–50nm	Pt, Pd, Au, Ru, Rh, Fe, Co, Ni, Cu, Al	[71]
Flash‐thermal shock synthesis	≈2073 K / 1 atm	≈mg level	<10 nm	Pt, Pd, Ru, Ir, Fe, Co, Ni, Cu, La, Ce, In Sr	[72]
Laser scanning ablation technology	‐ / 1 atm	≈mg level	2 nm	Pt, Ir, Cu, Ni, Cr	[99]
Wet chemical synthesis	≈453 K / 1 atm	≈mg level	5–10 nm	Pd, Pt, Ru, Rh, Ir	[51]
Wet chemical synthesis	443 K / high pressure	≈mg level	1–4 nm	Pt, Ir, Cu, In, Ga, Pb, Ni, Co, Fe	[100]
Electro chemical deposition synthesis	298 K / 1 atm	≈mg level	50–70 nm	Bi, Fe, Co, Ni, Mn	[88]
Sputtering deposition	298 K / vacuum	≈200mg	1–100nm	Ag, Pt, Ir, Ru, Co, Cr, Fe, Mn, Ni	[101]
Dealloying synthesis	‐ / 1 atm	≈g level	Porous bulk	Mn, Ni, Cu, Co, V, Fe, Mo, Cr, Pd Pt, Au, Ru, Ir, Ag, Rh, Os,	[95]

## 5 In Situ Transmission Electron Microscopy (In Situ TEM)

Upon examining the fundamental principles, rational design, and synthesis strategies for the formation of HEAs, we conclude that significant efforts have been made regarding these emerging materials; however, challenges still persist during the synthesis of HEAs. Therefore, researchers have acknowledged that a comprehensive understanding of synthesis processes and reaction mechanisms at the atomic and molecular levels is crucial for the design of advanced and high‐efficiency catalysts. In catalytic research, in situ TEM has become a vital technique compared to other in situ characterization methods such as X‐ray diffraction (XRD), X‐ray photoelectron spectroscopy (XPS), X‐ray absorption spectroscopy (XAS), Fourier‐transform infrared spectroscopy (FTIR), and Raman spectroscopy. While these spectroscopic techniques primarily provide average data under reaction conditions,^[^ ^102^, ^103^, ^104^ ^]^ in situ TEM allows direct observation of individual atoms or molecules during reactions.

Recent advancements in in situ characterization techniques, including environmental TEM (ETEM), liquid and gas phase electron microscopy methods, have made it possible to observe functional materials under real catalytic conditions.^[^ ^105^, ^106^, ^107^, ^108^ ^]^ Although it holds significant disruptive potential, the utilization of in situ TEM for HEA‐based catalysts is still largely unexamined, mainly due to the difficulties associated with multicomponent complexity, elevated temperature conditions, and swift reaction kinetics. The following section consists of two parts: first, we will discuss the current progress about understanding the growth mechanisms of HEAs under in situ TEM, and second, we will examine their structural and compositional evolution under real catalytic conditions in TEM.

### 5.1 Formation and Growth of HEA NPs Under In Situ TEM

**Figure 6:**
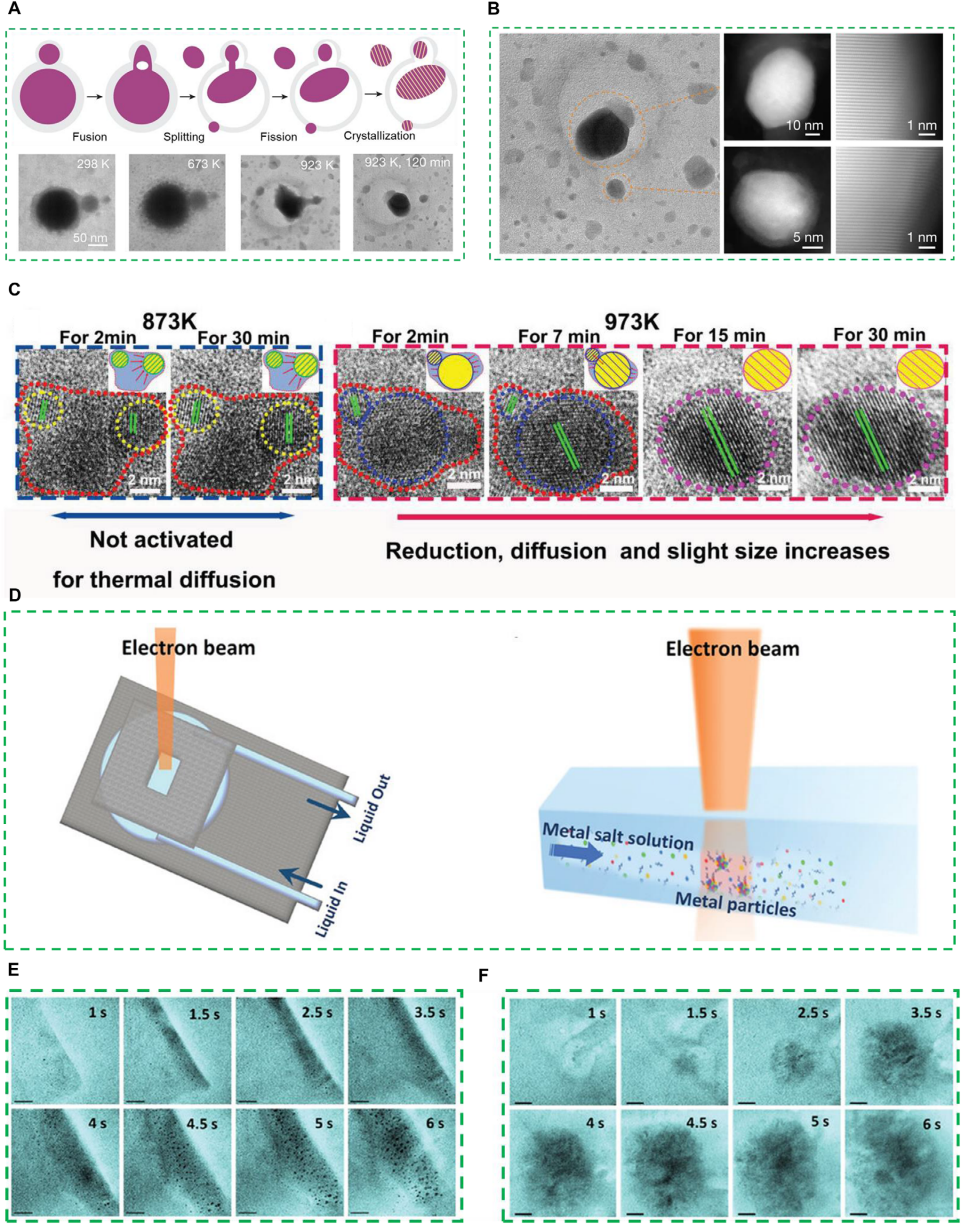
A) schematic diagram of the transition process and a series of In Situ TEM images B) showing the low and magnified HAADF‐STEM images. Reproduced with permission.^[^ ^26^ ^]^ Copyright 2023, Springer Nature. C) shows in situ TEM image sequences of HEA‐NPs‐(14) during heat treatment. Reproduced with permission.^[^ ^98^ ^]^ Copyright 2023, Wiley‐VCH GmbH. D) Schematic of the flowing liquid cell TEM holder and process of particle formation under the electron beam. E) Series of TEM snapshots showing the dynamic evolution of primary colloidal NPs as they form and grow within the liquid solution F) Series of TEM snapshots showing the dynamic formation and spread of a cloud‐like network of secondary colloidal NPs within the liquid solution. The scale bar is 50nm. Reproduced with permission.^[^ ^29^ ^]^ Copyright 2024, Wiley‐VCH GmBH.

In situ TEM has the capability to enable real‐time observation of materials under controlled environments (gas/liquid) and external stimuli (heat, electrical bias, light). This technique tracks atomic‐scale structural evolution, property changes, and growth mechanisms dynamically, providing insights inaccessible to conventional microscopy.^[^ ^109^ ^]^ For example. Fu et al.^[^ ^26^ ^]^ utilized in situ technique to unravel the formation mechanism of HEA‐NPs, capturing a striking fusion‐fission crystallization sequence mediated by liquid Ga (**Figure** **6**A). Their in situ ETEM studies revealed that Ga NPs first fused and coalesced with surrounding metal precursors at elevated temperatures (29.5–97.6 K), forming a metastable two‐domain structure connected by a thin channel at 923 K. This configuration then underwent abrupt fission (splitting at 5.32–5.46 s) due to surface tension gradients and gas release from precursor decomposition. Prolonged annealing at 923 K (30–120 min) drove crystallization, as confirmed by HRTEM lattice fringes and uniform elemental distribution in HAADF‐STEM (Figure 6B). These observations underscore liquid Ga's dual role as a mixing medium and dynamic template, which promotes atomic diffusion and strain relaxation during HEA‐NP formation.

Moreover, Li et al.^[^ ^98^ ^]^ employed in situ TEM to investigate the temperature‐dependent evolution of HEA‐NPs during heat treatment. Their observations revealed that at 873 K, the thermal energy was insufficient to drive atomic diffusion, leaving the precursor morphology intact even after 30 min. In contrast, at 973 K, rapid interdiffusion facilitated the formation of a single‐phase solid‐solution nanoalloy within just 15 min, with only minor particle coarsening during prolonged annealing (Figure 6C). This confirms 973 K as the optimal temperature for their step‐alloying approach. Furthermore, Yassar et al.^[^ ^29^ ^]^ employed in situ liquid‐cell TEM (Figure 6D) to investigate the colloidal synthesis pathways of multielement alloy nanoparticles (Au─Pt─Ir─Cu─Ni) under the electron beam. As demonstrated, the flowing precursor solution enabled direct observation of two distinct nucleation stages, 1) Primary particles (10–30 nm, Au/Cu‐rich) formed through rapid precipitation and growth, analogous to conventional batch reduction synthesis (Figure 6E) and 2) Secondary particles (<4 nm, Pt─Cu─Ir─Ni) that evolved via a colloidal network mechanism with delayed phase separation (Figure 6F). The study revealed that beyond thermodynamic miscibility, kinetic factors, including metal ion reduction potentials and oxidation states, critically govern elemental partitioning during early nucleation stages.

**Figure 7:**
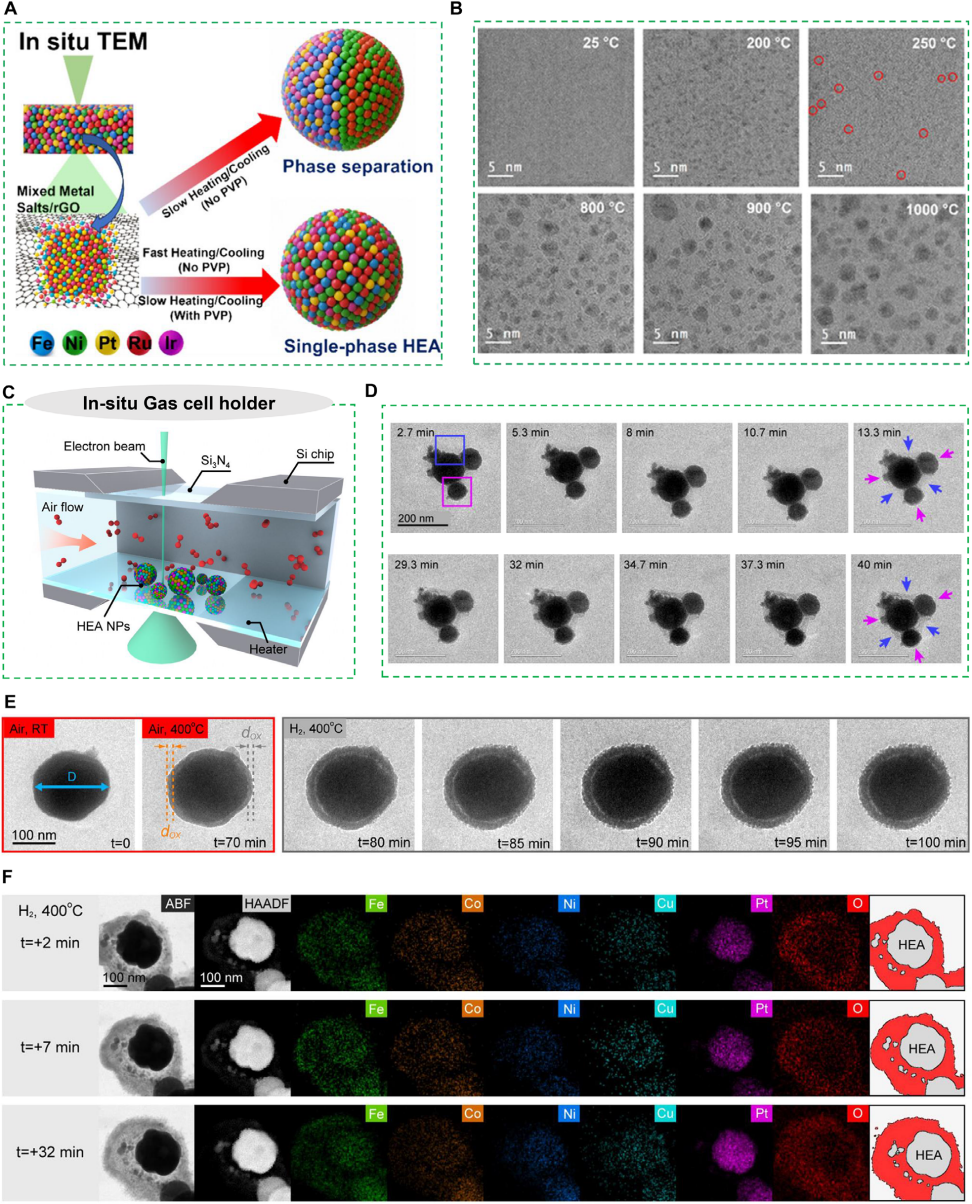
A) schematic illustration of HEA NPs forming on rGO nanosheets via in situ TEM, B) In situ HRTEM imaging of converting the mixed metal salt precursor film into NPs on rGO during slow heating and cooling process. Reproduced with permission.^[^ ^110^ ^]^ Copyright 2025, American Chemical Society. C) Representation of in situ gas cell. D) shows a series of in situ TEM images of HEA NPs during annealing in air. Reproduced with permission.^[^ ^111^ ^]^ Copyright 2020, Wiley‐VCH GmbH E) in situ TEM images showing HEA NPs oxidation in air (red box) and reduction in H_2 _(gray box). F) Corresponding reduction process with time stamps, ABF/HAADF images, EDS maps, and NPs oxide evolution. Reproduced with permission.^[^ ^112^ ^]^ Copyright 2021, American Chemical Society.

Recently, Saray et al.^[^ ^110^ ^]^ employed in situ aberration‐corrected TEM to unravel the nucleation and growth mechanisms of FeNiPtIrRu HEA NPs derived from mixed metal‐chloride precursors on reduced graphene oxide (rGO). By systematically varying thermal conditions (slow versus fast heating/cooling rates), they revealed how kinetics and thermodynamics govern phase evolution and elemental homogeneity (**Figure** **7**A). Under slow heating (20 °C min^−1^), the transformation began at ≈250 °C with the formation of crystalline nuclei (≈1 nm), followed by progressive nanoparticle growth via atomic incorporation and cluster coalescence (Figure 7B). At 1000 °C, the nanoparticles attained a single‐phase fcc structure, but slow cooling induced phase segregation due to enhanced atomic diffusion and thermodynamic immiscibility. In contrast, rapid heating/cooling (10^3^ °C s^−1^) kinetically trapped the elements in a metastable FCC solid solution, suppressing segregation. To mitigate segregation under slow thermal treatment, the authors introduced PVP into the precursor solution. In situ TEM revealed that PVP delayed nucleation (onset at ≈700 °C vs 250 °C without PVP) and restricted atomic diffusion, yielding smaller, homogeneous HEA NPs. The polymer‐derived carbon matrix acted as a physical barrier, stabilizing the high‐entropy state against phase separation. These real‐time observations underscore the power of in situ TEM in elucidating dynamic processes such as nucleation, coalescence, and elemental redistribution that are inaccessible via ex situ methods. By correlating thermal conditions with atomic‐scale structural evolution, this work provides a roadmap for tailoring HEA synthesis to achieve desired phase purity and catalytic performance.

### 5.2 In Situ TEM Investigation of HEAs Evolution Under Reaction Conditions

In situ TEM provides real‐time atomic‐scale visualization of HEAs catalysts under reaction conditions, capturing dynamic processes like surface reconstruction and elemental segregation. For example, Song et al.^[^ ^111^, ^112^ ^]^ leveraged in situ ETEM to unravel the oxidation and reduction behavior of FeCoNiCuPt HEA NPs, demonstrating the technique's unique capability to monitor dynamic processes in real time under reactive gas environments (Figure 7C). Their study revealed that oxidation in air at 400 °C proceeds via Kirkendall effects, with outward diffusion of transition metals (Fe > Cu > Co > Ni) forming a disordered oxide layer, while Pt remained confined to the core (Figure 7D). In situ STEM‐EDS combined with annular bright‐field (ABF) and HAADF imaging captured the evolution of oxide thickness for 100–130 nm NPs), showing size‐dependent heterogeneity smaller NPs developed uniform oxide layers, whereas larger NPs exhibited uneven growth. Notably, ETEM revealed void formation due to differential metal diffusion rates, with Fe exhibiting the fastest outward mobility.

The power of in situ ETEM was further highlighted during reduction under H_2_ at 400 °C. Real‐time imaging showed the oxide layer expanding into a porous structure, with CuOx fully reducing to metallic Cu, while Fe, Co, and Ni oxides persisted at the surface. The oxide thickness increased from ≈10 to ≈20 nm for smaller NPs (core shrinkage from ≈178 to ≈173 nm) and ≈50 to ≈70 nm for larger NPs, driven by concentration gradients that enriched transition metals at the oxide‐gas interface (Figure 7E,F). These observations, enabled by ETEM's atomic‐scale spatial and temporal resolution, uncovered a critical size dependence in redox kinetics and segregation behavior insights unattainable through ex situ methods. The study underscores ETEM's unparalleled ability to correlate morphological changes (e.g., void formation, core‐shell evolution) with compositional gradients under operando conditions, providing a roadmap for designing oxidation‐resistant HEAs.

**Figure 8:**
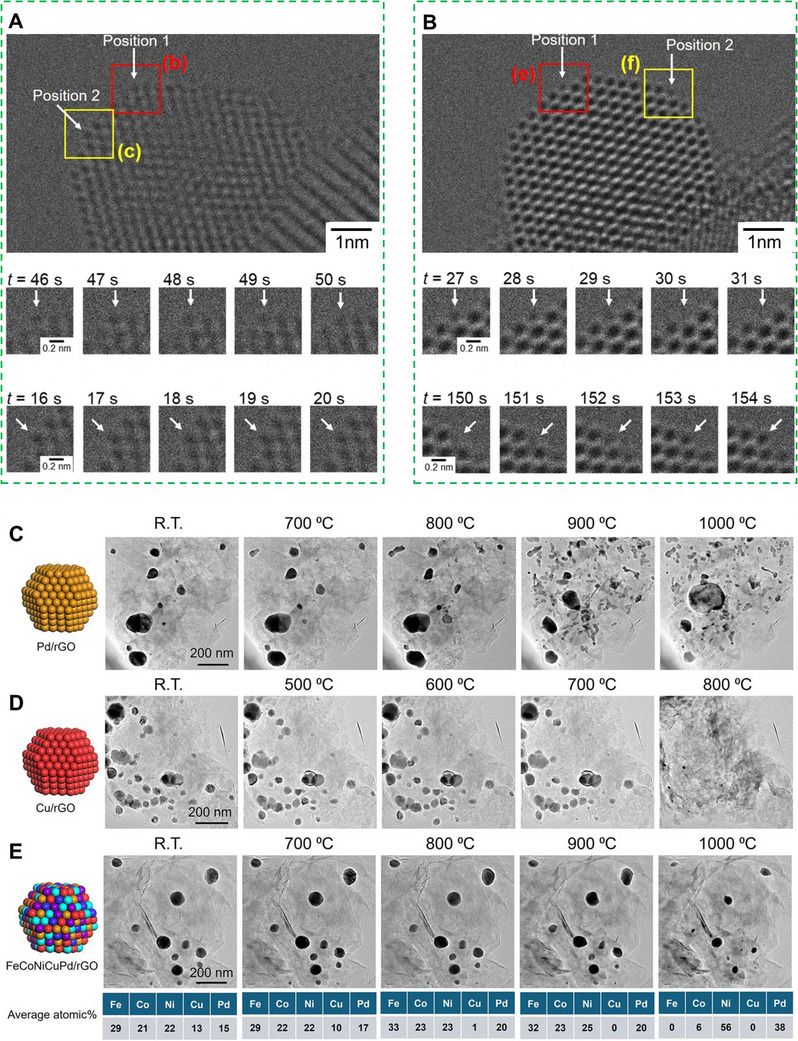
Representative TEM image along with sequential atomic‐resolution images from TEM movies of a nanoparticle in A) HEA/TiO_2_ and B) Pd/TiO_2_ under long‐time electron beam irradiation. Reproduced with permission.^[^ ^96^ ^]^ Copyright 2021, Springer Nature. Representative in situ TEM images of C) monometallic Pd, D) Cu, and E) quinary FeCoNiCuPd on rGO, as obtained between room temperature and 1000 °C. Reproduced with permission.^[^ ^113^ ^]^ Copyright 2024, American Chemical Society.

Moreover, Mori et al.^[^ ^96^ ^]^ reported that CoNiCuRuPd/TiO_2_ NPs exhibit remarkable robustness, where atomic column contrasts remained relatively stable even at the edge and corner regions during prolonged electron beam irradiation (**Figure** **8**A). In contrast, monometallic Pd/TiO_2_ showed pronounced contrast fluctuations, attributed to knock‐on damage and atomic displacement (Figure 8B). This direct visualization highlights how in situ TEM can effectively differentiate the resistance of multicomponent HEAs against structural deterioration compared to conventional monometallic systems, thereby underscoring the intrinsic stability of HEA catalysts under harsh conditions. More recently, Hashimoto et al.^[^ ^113^ ^]^ systematically evaluated the thermal stability of HEA NPs under in situ heating conditions. In monometallic systems such as Pd/rGO, NPs began to deform above 700 °C and underwent severe restructuring at 900 °C, resulting in bimodal size distributions (Figure 8C). Similarly, Cu/rGO NPs exhibited gradual shrinkage between 500–600 °C and completely disappeared by sublimation at 800 °C (Figure 8D). In sharp contrast, quinary HEA NPs on rGO displayed remarkable thermal robustness, maintaining their size, shape, and composition up to 700 °C with only gradual shrinkage at 800 °C, primarily due to Cu loss rather than agglomeration (Figure 8E). High‐resolution TEM further confirmed their structural integrity, revealing stable lattice fringes of the fcc structure. These findings clearly demonstrate that the high configurational entropy of HEAs contributes to their exceptional thermal stability and resistance to deterioration compared to conventional monometallic catalysts.

In conclusion, the unique capability of in situ TEM has been demonstrated to elucidate fundamental growth mechanisms in complex alloy systems. By providing atomic‐scale, time‐resolved visualization of transient intermediates and competing growth pathways, the technique offers unparalleled insights for rational design of HEAs. Such real‐time observations are particularly valuable for understanding composition‐structure relationships and tailoring synthesis protocols to achieve desired HEAs.

### 5.3 Current Limitations of In Situ TEM

While in situ TEM has emerged as a powerful tool to probe the dynamic structural evolution of HEA NPs during growth and catalysis, several intrinsic limitations remain. Beam‐induced effects such as heating, atom migration, and knock‐on damage can obscure intrinsic growth mechanisms, especially in chemically complex HEAs where element‐specific contrast is challenging to resolve.^[^ ^109^ ^]^ Moreover, current gas‐ and liquid‐cell TEM platforms still operate within restricted pressure and temperature windows, which limit the direct correlation to realistic catalytic environments.^[^ ^114^ ^]^ Quantitative extraction of reaction kinetics and diffusion parameters is further complicated by the interplay between the electron beam, the support, and the reactive medium

## 6 Conclusion and Future Outlooks

HEAs have emerged as a revolutionary category of materials owing to their exceptional structural and functional adaptability. To fully harness their potential, precise control over the synthesis that dictates composition, microstructure, and morphology is crucial. This review has examined the fundamental principles of HEAs, emphasized recent advancements in nanoscale synthesis techniques, and pinpointed ongoing challenges, such as attaining uniform elemental distribution, mitigating high‐temperature phase segregation, and coordinating the reduction of multiple precursors. Advancements in TEM have enhanced the understanding of the dynamic growth mechanisms of HEA nanoparticles.

1. Despite major progress, there are still key challenges. The complex nature of HEAs makes it difficult to design materials with specific properties. Future research should focus on making HEAs with controlled compositions, uniform sizes, specific shapes, and advanced structures. Understanding short‐range atomic arrangements, how elements interact at interfaces, and how they are distributed will be important for improving their performance. Combining non‐traditional synthesis methods with precise solution‐based techniques could help produce customized HEAs on a larger scale and with high efficiency
2. Moreover, the vast compositional space of HEAs necessitates a transition from empirical exploration to systematic design. High‐throughput computational modeling and machine learning can expedite the discovery of optimal elemental combinations and elucidate the synergistic in multicomponent systems.
3. A range of in situ characterization techniques beyond TEM provides complementary insights into catalytic processes, yet each comes with inherent limitations. For instance, in situ XAS reveals oxidation states and coordination environments with element specificity, but it lacks spatial resolution. Synchrotron‐based XRD tracks global phase transitions, yet cannot resolve the behavior of individual NPs. Neutron scattering is particularly powerful for probing light elements such as hydrogen and mapping diffusion pathways, though its temporal and spatial resolution are limited. Operando Raman and XPS add valuable vibrational and surface‐chemical information but cannot directly visualize atomic‐level dynamics. In contrast, in situ TEM uniquely captures structural evolution at near‐atomic resolution, directly imaging nucleation, growth, and transformation events that remain inaccessible to ensemble‐averaging techniques. Nevertheless, TEM observations often represent local behavior, making integration with complementary spectroscopic and scattering methods essential for achieving a holistic understanding. Looking ahead, progress will rely on integrating in situ TEM with advanced spectroscopic modalities (EELS, 4D‐STEM, operando MS/GC), machine‐learning‐assisted atomic tracking, and theoretical modeling to disentangle multielement dynamics. Innovations in MEMS‐based cells enabling higher‐pressure/high‐temperature operando conditions, together with low‐dose fast detectors, will be critical for faithfully capturing catalytic processes in HEAs. Recent advances in graphene‐based (GO/graphene) liquid‐cell TEM, particularly graphene hBN heterostructures, offer sub‐nanometer resolution, with engineered graphene liquid cells already achieving ≈1 nm STEM‐EDX imaging of bimetallic nanoparticles in water.^[^ ^115^ ^]^ Incorporating such GO‐enhanced windows will provide unprecedented insights into HEA nucleation under wet‐chemistry synthesis and reveal catalytic mechanisms under electrocatalytic conditions. Collectively, these advances will extend in situ TEM from model studies toward industrially relevant environments, guiding the rational design of robust, multi‐element nanocatalysts.

The integration of these technologies with automated synthesis and characterization will facilitate the discovery of application‐specific HEAs. The quest for regulated HEA synthesis signifies a cutting‐edge development in materials science. Interdisciplinary collaboration that integrates enhanced fabrication, real‐time characterization, and data‐driven design will advance the development of next‐generation HEAs for innovative technologies.

## Conflict of Interest

The authors declare no conflict of interest.
